# Quantification of narrow band UVB radiation doses in phototherapy using diacetylene based film dosimeters

**DOI:** 10.1038/s41598-020-80115-5

**Published:** 2021-01-12

**Authors:** Apoorva Mittal, Manoj Kumar, N. Gopishankar, Pratik Kumar, Akhilesh K. Verma

**Affiliations:** 1 Department of Medical Physics, Dr. B. R. A. Institute Rotary Cancer Hospital, All India Institute of Medical Sciences, New Delhi, 110029 India; 2 Department of Chemistry, University of Delhi, New Delhi, 110007 India

**Keywords:** Chemistry, Materials science

## Abstract

Narrow band ultraviolet B (NB UVB) radiation doses are administered during phototherapy for various dermatological ailments. Precise quantification of these doses is vital because the absorbed irradiation can cause adverse photochemical reactions which can lead to potential phototherapeutic side effects. The paper presents development of diacetylene based dosimeter for the determination of therapeutic NB UVB doses during phototherapy. The amide terminated diacetylene analogues have been synthesized by tailoring them with different functional groups. The synthesized diacetylene monomers have been introduced in a polyvinyl alcohol binder solution to obtain a film dosimeter. The influence of different headgroups on the colorimetric response to UV radiation has been studied. Among all the synthesized diacetylene analogues, the naphthylamine substituted diacetylene exhibited excellent color transition from white to blue color at 100 mJ cm^−2^ NB UVB radiation dose. The developed amide films can be easily pasted on multiple sites of the patient’s skin to monitor doses during phototherapy simultaneously at different anatomical regions. The digital image processing of the scanned images of the irradiated films facilitates rapid dose measurement which enables facile implementation of the developed film dosimeters and promising application in routine clinical dosimetry.

## Introduction

The sudden rise in the incidence of skin cancer has propelled the investigation of measurement methods of ultraviolet radiation (UVR) dosimetry to the forefronts^1–5^. The UVR output from natural sunlight and therapeutic artificial sources needs proper monitoring to protect the human skin from their toxic effects^6–10^. In hospitals, phototherapy for the treatment of skin diseases including psoriasis, mycosis fungoides, vitiligo and atopic dermatitis involves exposure to UVA (320–400 nm) and UVB (280–320 nm) component of ultraviolet radiation^11^. Lately introduced Narrow Band UVB (NB UVB 311–312 nm) phototherapy has shown improved efficacy in the alleviation of moderate-to-severe skin diseases and is a commonly used treatment modality^12–14^.

The benefits of phototherapy are accompanied with acute and chronic side effects on patient’s skin^15^. The acute side effects of excessive therapeutic UVR exposure during phototherapy include erythema i.e. skin reddening, pigment darkening and skin blistering^16,17^. Prolonged exposures result in epidermal thickening and degradation of keratinocytes which leads to premature skin aging. Exposure of the eyes to UVR can cause cataract and keritoconjuctivitis^18,19^. The local and systematic immunosupression feature of UVR contributes to the development of skin carcinoma^20–22^. Therefore, besides the advantages of phototherapy in clearing dermatoses it exhibits harmful effects on the patients. In order to minimize the adverse chronic and acute effects of UV doses in phototherapy without compromising the effectiveness of the treatment accurate dosimetry of therapeutic UVR is required. In this context, novel film dosimeters for the measurement and monitoring of NB UVB doses in phototherapy have been developed in this study. The whole-body phototherapy is administered in a chamber containing long fluorescent lamps that generate NB UVB radiation spectra^23^. In order to monitor the radiation doses from the phototherapy cabinet, hand-held UV radiometers are currently the most preferred option for the dosimetry of phototherapy units which measure the radiation output but with limited applicability. For monitoring the NB UVB radiation doses, the UV radiometer is placed at one location in the phototherapy cabinet when there is no patient and hence, the measurement does not show the actual radiation dose absorbed by the patient’s skin. The term dose (mJ cm^−2^) in context of phototherapy refers to the energy of UV radiation deposited on a unit surface area during irradiation at a specified period of time^24^. A single measurement of radiation doses recorded by UV radiometer does not necessarily display the net radiation dose received by a patient undergoing phototherapy at any given anatomical site. A number of radiometers would be required to measure the UV doses at multiple sites of patient’s body simultaneously. According to the guidelines of British Dermatology Group, dosimetry using hand-held radiometer is a direct method which involves exposure to the occupant of the cabin while making measurements^25^. Also, in order to determine the radiation doses from phototherapy cabinet accurately, a large number of measurements must be integrated which puts the dosimetrist at the risk of significant UV radiation exposure^26^. In order to authenticate the efficacy of phototherapy cabinets, improved dosimeters are required for the evaluation of variation in administered radiation dose irradiated from phototherapy unit and the radiation dose that actually impinges on the patient’s skin. Owing to these issues, film dosimeters were developed in the present work which can be easily pasted on the patient’s skin in large number during radiation exposure.

Diacetylenes (DA) are an intriguing class of conjugated polymers which exhibit distinct color change in response to various external stimuli. The existence of delocalized π-electron networks and conformational restrictions offer interesting optical, structural and spectral features. These features can be manipulated by customizing the side chain of the diacetylenes with different head groups. A diverse variety of applications have been extensively studied ever since the first report by Wegner^27–31^. However, their application as film dosimeter for UVR dosimetry in NB UVB phototherapy for the patients having dermatological ailments has not been explored yet. Accordingly, the rationale of this work is to develop a novel and clinically viable NB UVB film dosimeter. Amide substituted DA derivatives were synthesized which exhibit significant response to low and high range of therapeutic NB UVB doses. The synthesized DA monomers were used to prepare film dosimeters by introducing them in a suitable binder to illustrate their utility in clinical applications.

## Results

### Synthesis of diacetylene monomers

**Figure 1:**
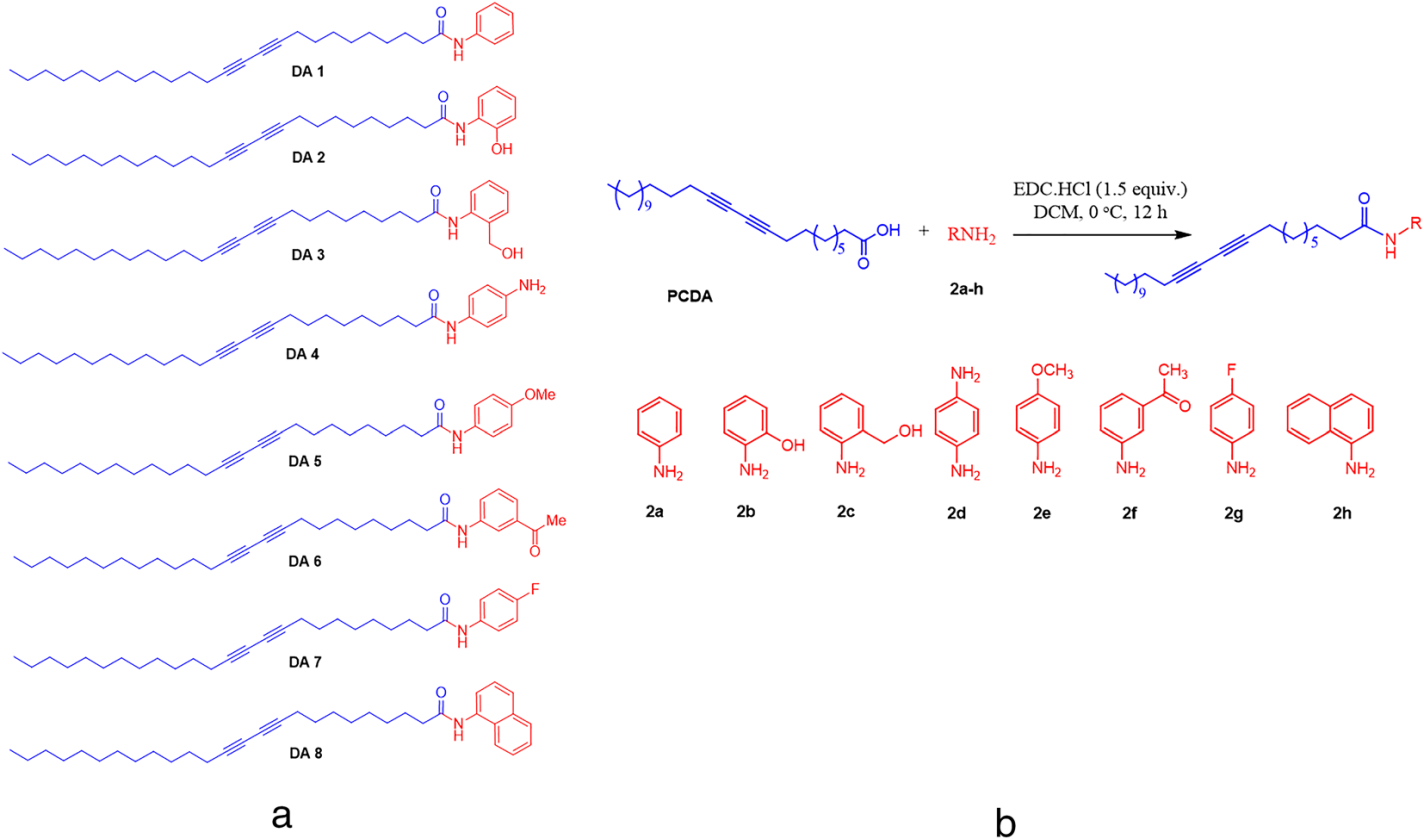
(**a**) Structure of the synthesized diacetylene derivatives DA 1–8. (**b**) Reaction scheme for the synthesis of diacetylene monomers.

10, 12 Pentacosadiynoic acid (PCDA) was chosen as the template molecule and various aminating reagents were substituted to compare the radiation sensitivity trends of the synthesized compounds. The diacetylene monomers DA 1–8 (Fig. 1a) used in this study were synthesized by single-step conversion of diacetylene carboxylic acids into amides. The amides were prepared by treating the carboxylic acid of diacetylene with substituted amides in the presence of ethyldimethylaminopropylcarbodiimide hydrochloride (EDC.HCl) which acts as a coupling reagent. The overall reaction lead to the formation of the amide. Figure 1b shows the general reaction scheme and structure of various synthesized DA monomers. The synthesized compounds were characterized by Nuclear Magnetic Resonance (NMR) and High Resolution Mass Spectroscopy (HR-MS) (“Supplementary Information”).

### Preparation of DA based film dosimeter

The synthesized amide substituted diacetylene monomers were introduced in a solution of polyvinyl alcohol in water which acts as a binder. A homogenous emulsion was prepared to coat it on a polyethylene terephthalate (PET) substrate using automatic film applicator. Thick, uniform and sturdy film dosimeters were obtained which can be cut into any shape and pasted on the patient’s skin. The developed film dosimeter provides an effective means of integrating UVR exposure at numerous sites of patient’s skin simultaneously which can be inaccessible to the conventional bulky and expensive dosimeters. The immediate white to blue color transition induced by NB UVB radiation in the film dosimeters renders an attractive intrinsic feature in the development of processing-free direct visual dosimeters. After exposure, using digital image analysis all the film dosimeters were rapidly scanned and analyzed at once.

### NB UVB radiation induced changes in spectral characteristics

**Figure 2:**
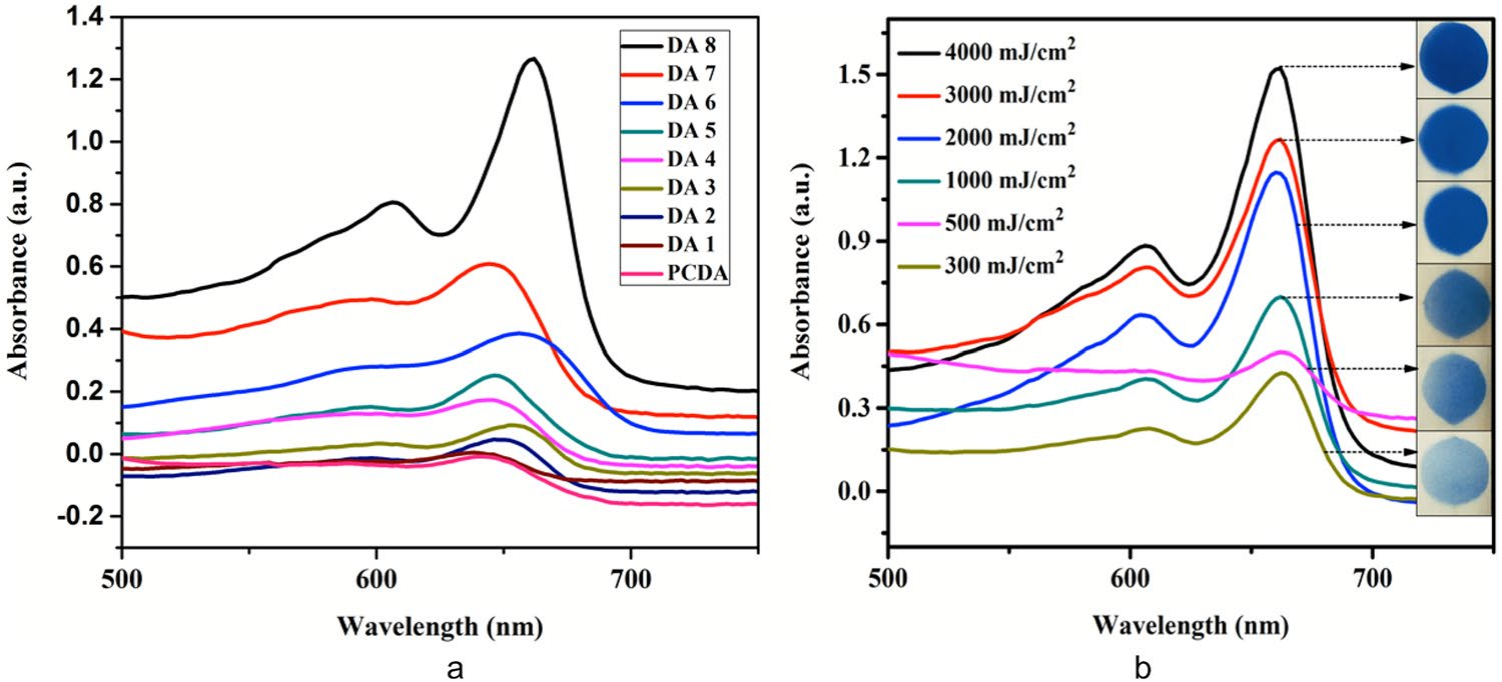
(**a**) Visible spectra of PCDA and DA 1–8 film dosimeters. (**b**) Visible absorption spectra of DA 8 film dosimeter.

NB UVB radiation induced photopolymerization of DA based film dosimeters resulted in blue colored polydiacetylenes which displayed an increase in the intensity of blue coloration with the NB UVB radiation doses. Figure 2a shows the visible absorption spectra of PCDA and DA 1–8 film dosimeters after exposure to NB UVB radiation. It is evident from the spectra that DA 8 displays the maximum absorbance thus; it is most sensitive to NB UVB radiation. A sensitivity order of DA 8 > DA 7 > DA 6 > DA 5 > DA 4 > DA 3 > DA 2 > DA 1 > PCDA can be inferred from the visible absorption spectra. The absorption spectra were acquired at various doses of NB UVB radiation to quantify the amount of coloration with respect to doses. The dosimeters were exposed within the dose limits followed in the protocol of NB UVB phototherapy^32^. Figure 2b shows the absorption spectra of the film dosimeters prepared with DA 8 and exposed to various NB UVB radiation doses ranging from 300 to 4000 mJ cm^−2^. A maximum absorption wavelength (λ_max_) at 660 nm can be seen with a shoulder peak at 600 nm. It is evident from the spectra that as the NB UVB radiation doses increase there is a concomitant increase in the absorbance peak of blue color at 660 nm. The absorption peak at 660 nm is due to the electron delocalization within the conjugated backbone, which appears visually as blue color. These spectral changes well corroborate with the visual observation as shown in photographic images. Previous studies suggest that the colorless to blue color transition is attributed to the changes in orientation, conformation and packing caused by the propagation of polydiacetylene chains. The headgroup hydrogen bonding also influences the coloration and susceptibility of DA monomer to change color. A lot of research work has been carried out to understand the mechanism of chromatic transitions however, it has proved to be a major challenge and the exact mechanism is still not completely known^33^.

**Figure 3:**
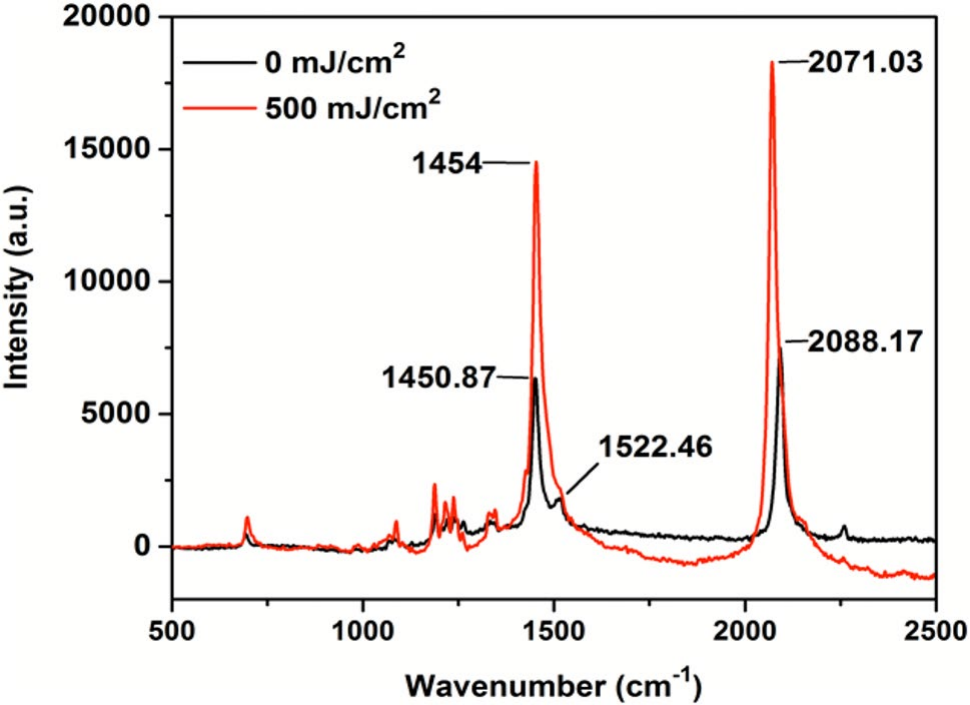
Raman spectra of DA 8 film dosimeter.

**Figure 4:**
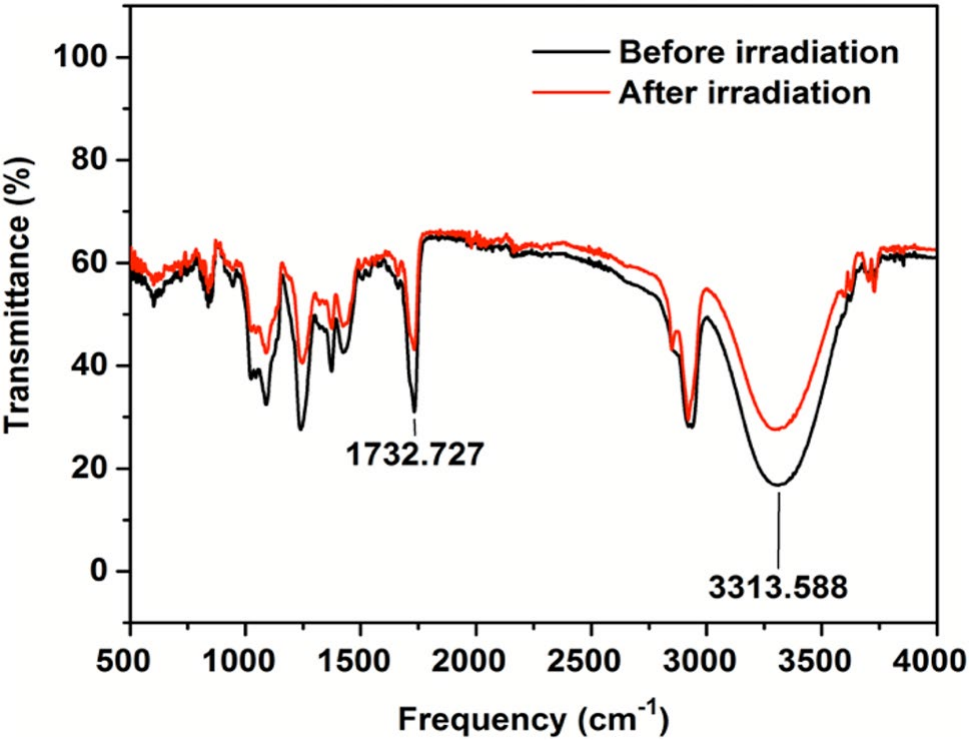
FTIR-ATR of film dosimeter DA 8 before and after NB UVB radiation exposure.

Fourier Transform Infrared-Attenuated Total Reflectance (FTIR-ATR) spectra of the DA 8 film dosimeter was acquired before and after exposure to NB UVB radiation (Fig. 3). The spectra show a broad peak at 3313 cm^−1^ which confirms the presence of –NH bond due to amide functionalization of the diacetylene monomer. The peak corresponding to amide linkage in the compound is also seen at 1732 cm^−1^. Post radiation exposure a decrease in the transmittance of the film dosimeter can be noticed which is due to the increase in the blue coloration in the film. In order to account for the variations in the bonding of diacetylene monomers upon exposure to radiation, Raman scattering analysis was carried out. Figure 4 shows the Raman spectra of DA 8 film dosimeter before and after irradiation to 500 mJ cm^−2^ NB UVB radiation dose. The presence of peaks at 1450 cm^−1^ and 2095 cm^−1^ is attributed to the characteristic conjugated ene–yne bands of carbon–carbon double bonds and carbon–carbon triple bonds respectively^34^. After radiation exposure, a slight shift in the ν (C=C) peak position from 1450 to 1454 cm^−1^ and ν (C≡C) peak position from 2088 to 2071 cm^−1^ can be noticed. This shift in the peak position can be attributed to the change in the conjugation length of the diacetylene monomer due to the effect of radiation. A significant increase in the intensity of the peaks is also noticed which is due to an overall increase in the hydrogen bonding of the system. The data from Raman spectroscopy also substantiates the finding that even low dose of NB UVB radiation (500 mJ cm^−2^) leads to considerable amount of polymerization which consequentially causes intense chromatic changes.

### NB UVB radiation induced changes in morphology in diacetylene monomer

**Figure 5:**
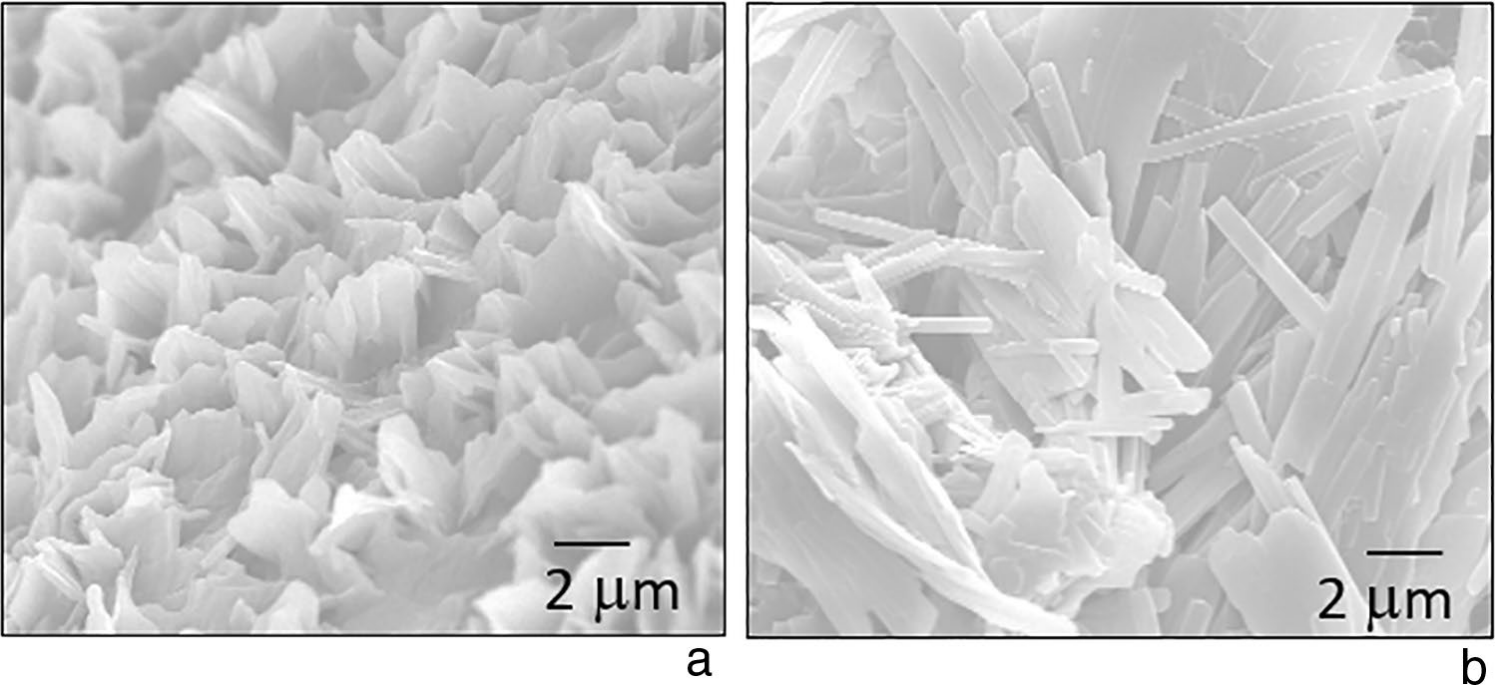
FESEM images of DA 8 (**a**) pre-irradiation (**b**) post irradiation to NB UVB dose.

In the Field Emission Scanning Electron Microscope (FESEM) image analysis of the synthesized diacetylene monomer DA 8 (Fig. 5a) densely populated ensemble of particles was observed before radiation exposure. Upon exposure to NB UVB radiation (Fig. 5b) a closely spaced thin filament-like morphology of the particles can be seen. During the polymerization reaction of the diacetylene, the side chain imposes a mechanical strain on the backbone due to the geometric constraints generated by intermolecular hydrogen bonding or Van der Waals interactions between side chains^35^. This mechanical strain could be responsible for the change in the morphology of the particles.

### Digital image analysis for practical application

**Figure 6:**
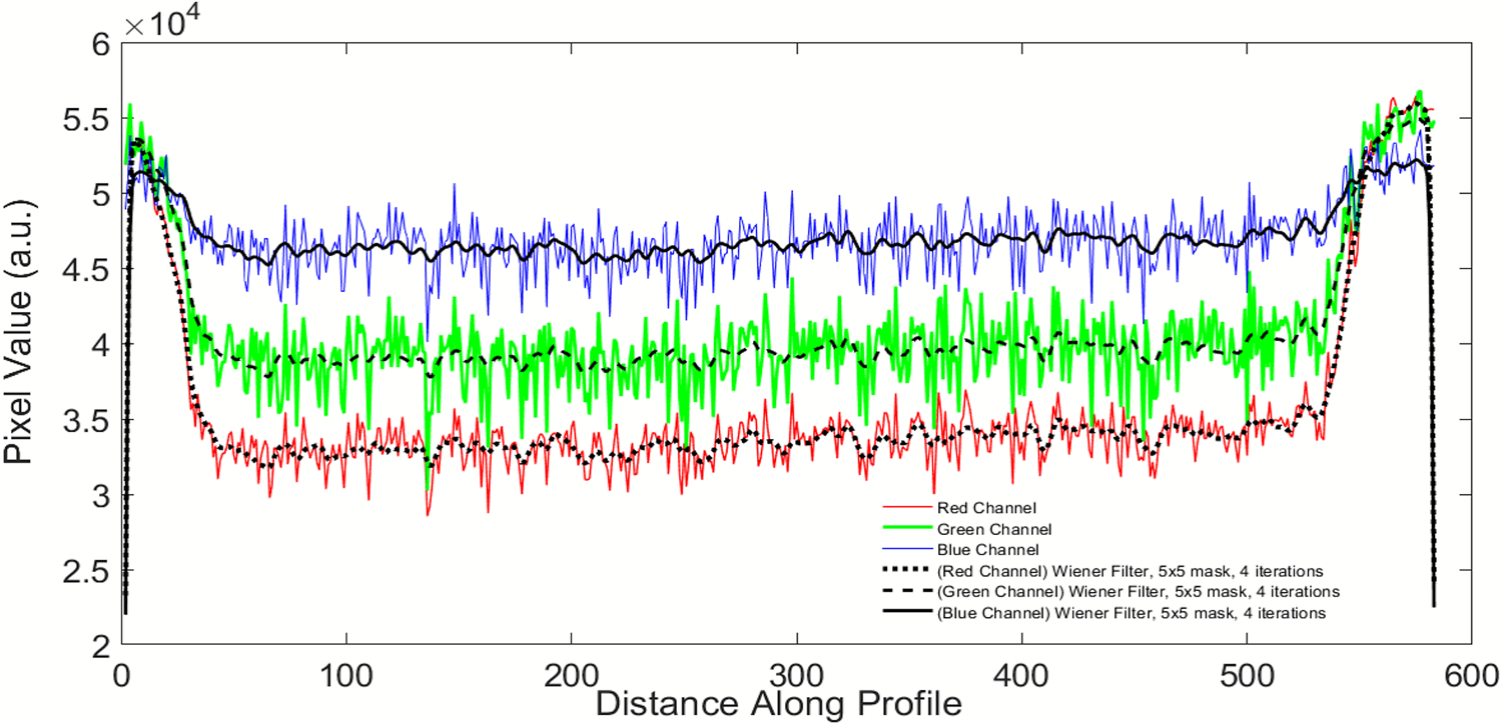
Pixel values in red, green and blue color channels of scanner.

The quantification of colorimetric dose response of the developed dosimeter was done by the measurement of the 2-D optical density (OD) using Epson 10000XL flatbed scanner^36^. Digital image analysis was carried out by scanning the dosimeters separately in each channel i.e. Red (R), Green (G) and Blue (B) thereby extracting the RGB channel values for each pixel within the dosimeters in the scanned images. (Fig. 6) The effect of background light was subtracted by taking white background and Weiner filter was applied to subtract the inherent noises of the scanner.

**Figure 7:**
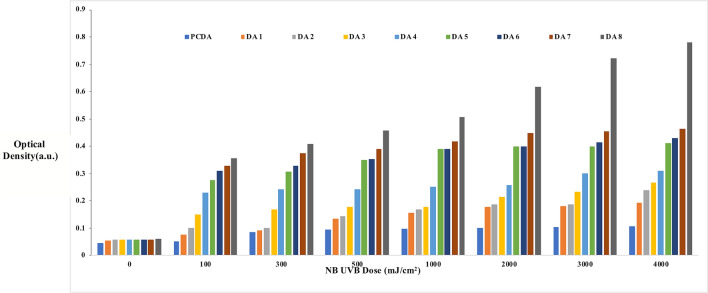
Digital image analysis of the prepared film dosimeters.

Since the absorption peak of the dosimeter due to the blue color of the polymerized compound falls in the red part of the spectrum, the red channel is more sensitive to variation in the optical density with NB UVB dose. Thus, red channel was chosen to monitor the changes in optical density with respect to NB UVB dose. The uniform dose distribution across the surface of the film dosimeter was confirmed by the RGB line profile of pixel values along the length of the DA 8 dosimeter. An in-house program was developed in MATLAB software to evaluate the changes in the amount of coloration due to NB UVB exposure by measuring the optical density for all the film dosimeters. Figure 7 represents the variation in optical density with NB UVB doses of the developed film dosimeters obtained after digital image analysis. The radiation sensitivity order was found to be in good correspondence with visual detection of chromatic transitions and visible spectroscopy as discussed previously.

**Figure 8:**
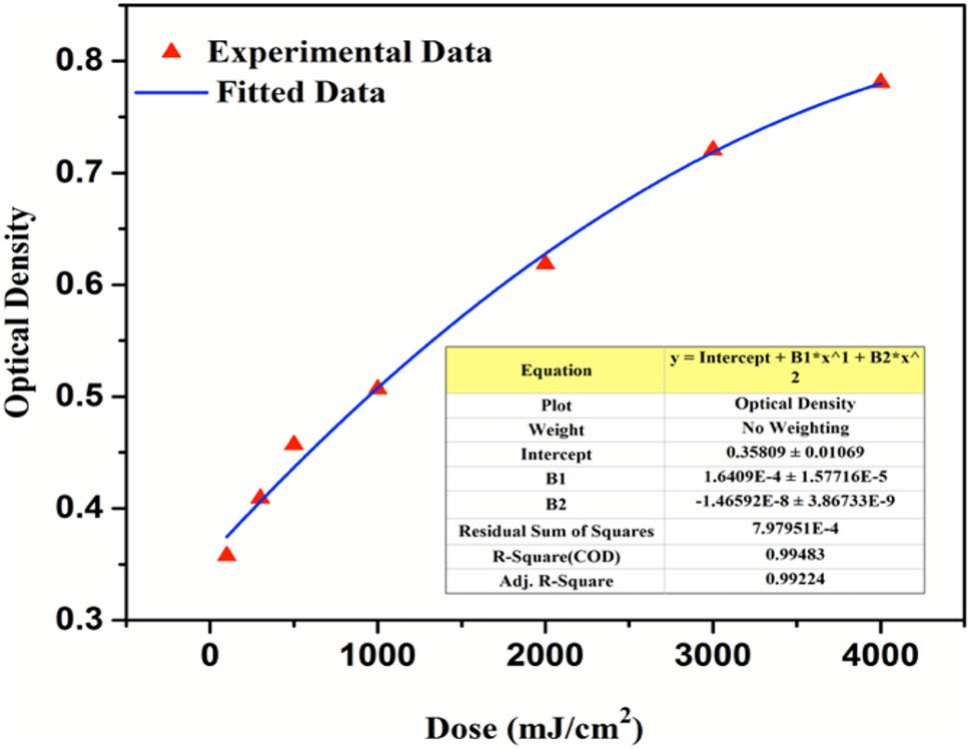
Calibration curve for DA 8 film dosimeter.

A calibration curve was plotted to quantify the radiation exposure per unit coloration of the film. Figure 8 represents the calibration curve between optical density versus known radiation dose for DA 8 dosimeter. The calibration function was generated by 2nd order polynomial fitting of the dataset. The calibration curve has linear or nearly linear response low to medium dose region and a non-linear high dose region. The sub-linearity at high dose signifies the dosimeter system approaching saturation which implies that at high radiation doses the difference in the optical density narrows. The linear region is most useful for the quantization of film sensitivity in terms of OD per unit absorbed dose. The established experimental calibration function can be used to generate a dose response curve where dose is an unknown parameter. The uncertainty in optical density measurement defined as the standard deviation of the mean value of optical density was evaluated by the statistical analysis of a series of measurements^37^. The mean and standard deviation were computed over a set of averaged measured intensities over the entire surface of ten unexposed and exposed films read five times each. The statistical uncertainty in optical density was obtained to be 1.8%. The accuracy of the film dosimeters was determined by comparing the dose response with standard radiometer readings. An accuracy of 1.5% was obtained in the dose response of the developed film dosimeters. Thus, by digital image analysis it can be concluded that the developed film dosimeters can be used for monitoring and measurement of NB UVB radiation doses from phototherapy cabinet in a rapid and cost-effective manner.

### Effect of various functional groups on radiation response

Upon undergoing topochemical polymerization, the DA units form conjugated polydiacetylenes (PDA) comprising ene–eye bonds which possess intense blue color. The topochemical polymerization is governed by a specific molecular arrangement of the monomers^38^. Typically, a distance of ~ 4.9 Å and an angle of 45° between the monomers is required for a successful topochemical reaction. The incorporation of the side groups on the DA main chain can bring the DA moiety into the desired reactive position leading to propagation of monomers and more efficient polymerization^39^. Also, the stress induced by the orientation and packing of the side groups on the DA backbone has significant influence on the electronic, optical and various physical properties of the monomers. In the present study, the role of different amide based functional groups on the NB UVB radiation induced color change has been studied. The 10,12 Pentacosadiynoic acid (PCDA) was taken as the main DA moiety whereby its acidic group was substituted by various amide headgroups. Particularly, amide containing headgroups were chosen because of the presence of an extra lone pair which is responsible for the enhanced hydrogen bonding which leads to improved radiation response of the DA monomers. The amide substituted film dosimeters exhibited better colorimetric response to radiation as compared to pure PCDA. This can be attributed to the presence of strong intermolecular hydrogen bonding in the amide containing PCDA monomers. Also, the intramolecular hydrogen bonding of the carbonyl group of PCDA with the amide hydrogen leads to improved blue coloration in the film dosimeters upon exposure to radiation. DA 8 shows the most visually significant blue coloration upon exposure to NB UVB dose of 100 mJ cm^−2^. This excellent color transition is supposedly due to the enhanced conjugation and aromaticity which increases π–π stacking leading to efficient topochemical polymerization reaction. The presence of highly electronegative fluorine group in DA 7 and ketone group in DA 6 enables stronger hydrogen bonding which leads to intense color change. Due to the lone pair conjugation of NH_2_ functional group in DA 4, it shows delocalization in the ring which allows improved hydrogen bonding thus leading to moderate color changes. Similarly, in DA 2 the phenoxide ion delocalizes in the aromatic ring. It is interesting to note that due to the presence of an extra carbon atom between the phenyl ring and –OH group in DA 3 the resonance of lone pair of oxygen with phenyl ring is quenched which consequently, exhibits better radiation sensitivity as compared to DA 2. In contrast to other headgroup substituted monomers, DA 5 develops lesser hydrogen bonding because of the presence methyl protected hydroxy group. The electron neutral aniline substituted DA 1 exhibits the least coloration in response to NB UVB radiation due to the absence of any heteroatomic group. It can be concluded that the incorporation of suitable substituent headgroups on the DA main chain can promote polymerization. Also, multiple hydrogen bonding and aromatic interactions of the substituted functional groups have pronounced effect on UV induced polymerization of the DA main chain which leads to chromatic transitions^40^. This understanding of the role of various functional groups on the colorimetric transitions led to the development of the most suitable film dosimeter for clinical dosimetry and quality assurance of the radiation source.

## Discussion

The DA based amide dosimeter fabricated in this paper enables visual detection and measurement of low to high range of therapeutic NB UVB radiation doses administered to the patients during phototherapy. By leveraging the ability of DA to undergo UVR induced polymerization which leads to chromatic transitions, a series of DA compounds were synthesized and coated to fabricate film dosimeter. The effect of variation of head group on the UV sensitivity was studied. Among all the amides, the naphthylamine substituted diacetylene exhibited immediate, significant coloration from white to blue upon exposure to 100 mJ cm^−2^ NB UVB radiation. Detection can be simply made by color transition from white to different intensities of blue coloration developed upon exposure to radiation dose delivered. The amount of coloration was quantified by studying change in its optical density using high resolution film scanner. The current investigation has led to the development of a potential clinically viable NB UVB dosimeter. With the perspective of clinical applications, several film dosimeters of different shapes and sizes can be pasted on the patient’s body and analyzed simultaneously. This could be a rapid tool to investigate if a phototherapy unit delivers the required doses. The dose received at the skin surface can be recorded to guide subsequent treatment doses as well as for the quality assurance of the phototherapy cabinet. However, a consolidate quality assurance procedure in UVR dosimetry needs to be designed using the developed film dosimeter.

## Experimental section

### General information

10, 12 Pentacosadiynoic acid (PCDA) was purchased from Sigma Aldrich and 1-Ethyl-3-(3-dimethylaminopropyl) carbodiimide hydrochloride (EDC.HCl) and was purchased from TCI Chemicals. ^1^H NMR (400 MHz) and ^13^C NMR (100 MHz) spectra were recorded in CDCl_3_. Chemical shifts for protons and carbons are reported in ppm from tetramethylsilane and referenced to the carbon resonance of the solvent. High resolution mass spectra (HR-MS) were recorded on electrospray mass spectrometer using positive electrospray ionization mode. UV visible absorption spectra of the film dosimeters was recorded using a Perkin Elmer Lambda 1050 spectrophotometer equipped with a 150 mm integrating sphere with 8° reflectance. FESEM images of diacetylene monomers studied here were obtained on Carl Zeiss (Gemini SEM 500). Freshly prepared compounds were deposited on silicon wafers. Samples were sputter coated on Quorum pure Au target, sputter time was 60 s and sputter current was 60 mA. Horiba LabRam HR revolution Raman spectrometer with 785 nm laser 50 mW power was used to record Raman spectra of the developed film dosimeters. The acquisition time was 2 s. FTIR spectra of the film dosimeters were obtained from Thermo Scientific spectrometer in ATR mode.

### Synthesis of diacetylene monomers

In an oven-dried round bottom flask, 10,12 Pentacosadynoic acid (PCDA) 1.0 equivalent in dichloromethane (DCM) was added dropwise in solution of EDC.HCl 1.5 equivalent in DCM at 0 °C. After 30 min, the substituted amide 1.0 equivalent was added to the reaction mixture. The resulting reaction mixture was run at room temperature for overnight. The progression of the reaction was monitored by TLC analysis; after complete consumption of starting material. The reaction mixture was diluted with DCM (10 mL) and water (15 mL). The layers were separated, and the organic layer was washed with brine solution and dried over sodium sulphate. Organic layer was concentrated under reduced pressure. The crude material so obtained was purified by column chromatography on silica gel (100–200) (hexane:ethyl acetate; 70/30). Other diacetylene monomers were also prepared by employing similar procedures. Spectroscopic data of the synthesized monomers is given in the supporting information.

### Fabrication of diacetylene monomer film dosimeter

A 5% aqueous solution of Polyvinyl Alcohol (PVA) was prepared. The synthesized DA monomer dissolved in ethyl acetate was added to the PVA solution in 1:1 ratio. The solution was stirred at 2000 rpm and heated at 60 °C for 3 h to form a homogenous turbid emulsion. The emulsion was coated on a transparent polyethylene terephthalate (PET) sheet using an automatic film applicator and left for drying overnight at room temperature. The thickness of the dried layer of the obtained films was 100 μm.

### Irradiation with whole-body NB UVB phototherapy cabinet

The samples were exposed in Waldmann UV Therapy System 7002. This whole-body phototherapy system comprises of 2 m long 42 TL 01 120 W narrow band UVB lamps for homogenous irradiation from head to toe. The phototherapy cabinet is equipped with TFT monitor with 10.4″ touchscreen where the doses can be programmed. The samples were kept at the centre and a range of doses were irradiated according to the phototherapy protocol for patients.

### Image digitalization

All the samples were scanned by using Epson 10000XL flatbed scanner in reflective mode. The images were digitalized in 48 bits RGB format at a resolution of 72 dpi and saved in uncompressed TIFF format. The image processing was performed using an open source program written in MATLAB 7.4. The analysis was performed in the red channel component of the image.

## Supplementary Information

Supplementary Information.
