# Supplementary material for: Quantification of narrow band UVB radiation doses in phototherapy using diacetylene based film dosimeters
Source: Sci Rep. 2021 Jan 12;11:684. doi: 10.1038/s41598-020-80115-5 (PMC7804282; doi:10.1038/s41598-020-80115-5)

Quantification of Narrow Band UVB Radiation Doses in Phototherapy Using Diacetylene Based Film Dosimeters

Apoorva Mittal[1], Manoj Kumar[2#], N. Gopishankar[1#], Pratik Kumar[1*] and Akhilesh K. Verma[2*]

[1] Department of Medical Physics, Dr. B. R. A. Institute Rotary Cancer Hospital, All India Institute of Medical Sciences, New Delhi 110029, India

[2] Department of Chemistry, University of Delhi, Delhi 110007, India

Spectroscopic data of synthesized monomers:

N-Phenylpentacosa-10,12-diynamide (DA 1)

^{1}H NMR (400 MHz, $CDCl_3$) δ 7.48 (d, *J* = 7.8 Hz, 3H), 7.24 (s, 2H), 7.07-7.00 (t, 1H), 2.28 (d, *J* = 7.8 Hz, 2H), 2.18 (t, *J* = 6.9 Hz, 4H), 1.71-1.60 (2H), 1.44 (q, *J* = 4.0 Hz, 4H), 1.20-1.31 (m, 26H), 0.83 (t, *J* = 6.6 Hz, 3H); ^{13}C NMR (101 MHz) δ 171.78, 138.15, 129.00, 124.21, 119.96, 77.74, 77.58, 65.43, 65.35, 37.80, 32.01, 29.73, 29.58, 29.45, 29.28, 29.20, 28.99, 28.96, 28.84, 28.45, 28.36, 25.71, 22.79, 19.28, 19.27, 14.23. HRMS (ESI-TOF) $[M+H]^+$ Calcd for $[C_{31}H_{48}NO]$ 450.3730, found 450.3727.

N-(2-Hydroxyphenyl)pentacosa-10,12-diynamide (DA 2)

^{1}H NMR (400 MHz, $CDCl_3$) δ 7.07-7.11 (m, 1H), 7.01 (d, *J* = 7.7 Hz, 1H), 6.63-6.67 (m, 2H), 5.76 (s, 1H), 2.22 (t, *J* = 6.9 Hz, 4H), 2.15 (t, *J* = 7.6 Hz, 2H), 1.60 (t, *J* = 7.1 Hz, 2H), 1.48 (dd, *J* = 14.7, 7.0 Hz, 4H), 1.21-1.37 (m, 26H), 0.87 (t, *J* = 6.8 Hz, 3H); ^{13}C NMR (101 MHz) δ 173.61, 145.74, 130.63, 129.33, 121.99, 117.67, 115.72, 77.73, 77.54, 65.39, 65.29, 49.21, 40.85, 36.73, 34.04, 32.00, 29.72, 29.56, 29.43, 29.19, 28.95, 28.79, 28.43, 28.35, 25.68, 25.03, 22.78, 19.29, 14.21 HRMS (ESI-TOF) $[M+H]^+$ Calcd for $[C_{31}H_{48}NO_2]$ 466.3680, found 466.3682.

N-(2-(Hydroxymethyl)phenyl)pentacosa-10,12-diynamide (DA 3)

^1H NMR (400 MHz, $CDCl_3$) δ 8.66 (s, 1H), 8.01 (d, J = 8.1 Hz, 1H), 7.29-7.34 (m, 1H), 7.18 (d, J = 6.9 Hz, 1H), 7.08 (t, J = 7.3 Hz, 1H), 4.67 (s, 2H), 3.00 (s, 1H), 2.36 (t, J = 7.6 Hz, 2H), 2.25 (t, J = 6.9 Hz, 4H), 1.68-1.75 (m, 2H), 1.49-1.56 (m, 4H), 1.25-1.43 (m, 26H), 0.90 (t, J = 6.8 Hz, 3H); ^{13}C NMR (101 MHz) δ 172.24, 137.47, 129.96, 129.09, 128.94, 124.38, 122.66, 77.71, 77.54, 65.40, 65.31, 64.42, 37.95, 32.00, 29.72, 29.57, 29.43, 29.24, 29.19, 29.01, 28.95, 28.85, 28.44, 28.38, 25.73, 22.78, 19.28, 14.22. HRMS (ESI-TOF) $[M+H]^+$ Calcd for $[C_{32}H_{50}NO_2]$ 480.3836, found 480.3862.

N-(4-Aminophenyl)pentacosa-10,12-diynamide (DA 4)

^1H NMR (400 MHz, $CDCl_3$) δ 7.23-7.25 (m, 2H), 7.13 (s, 1H), 6.59-6.63 (m, 2H), 2.28 (t, J = 7.6 Hz, 2H), 2.21 (t, J = 6.9 Hz, 4H), 1.64-1.71 (m, 2H), 1.45-1.52 (m, 4H), 1.22-1.34 (m, 26H), 0.84-0.88 (m, 3H); ^{13}C NMR (101 MHz) δ 171.30, 143.28, 129.43, 122.07, 115.48, 77.72, 77.58, 65.37, 65.31, 37.65, 32.00, 29.71, 29.57, 29.43, 29.27, 29.18, 28.98, 28.94, 28.84, 28.43, 28.36, 25.78, 22.77, 21.14, 19.28, 14.27, 14.22. HRMS (ESI-TOF) $[M+H]^+$ Calcd for $[C_{31}H_{49}N_2O]$ 465.3839, found 465.3847.

N-(4-Methoxyphenyl)pentacosa-10,12-diynamide (DA 5)

^1H NMR (400 MHz, $CDCl_3$) δ 7.68 (s, 1H), 7.38 (d, J = 8.8 Hz, 2H), 6.78 (d, J = 8.8 Hz, 2H), 3.74 (s, 3H), 2.27 (t, J = 7.4 Hz, 2H), 2.20 (t, J = 6.5 Hz, 4H), 1.64 (d, J = 6.6 Hz, 2H), 1.47 (d, J = 5.6 Hz, 4H), 1.27 (d, J = 37.5 Hz, 26H), 0.83-0.87 (m, 3H); ^{13}C NMR (101 MHz) δ 171.71, 156.32, 131.32, 121.92, 114.07, 77.70, 65.43, 65.36, 55.49, 37.54, 32.00, 29.72, 29.57, 29.43, 29.29, 29.18, 29.00, 28.94, 28.85, 28.44, 28.37, 25.78, 22.77, 21.12, 19.27, 14.22. HRMS (ESI-TOF) $[M+H]^+$ Calcd for $[C_{32}H_{50}NO_2]$ 480.3836, found 480.3848.

N-(3-Acetylphenyl)pentacosa-10,12-diynamide (DA 6)

^{1}H NMR (400 MHz, $CDCl_3$) δ 10.46 (s, 1H), 8.34 (d, J = 8.5 Hz, 1H), 8.08 (dd, J = 5.0, 1.0 Hz, 1H), 7.71-7.76 (m, 1H), 7.02 (ddd, J = 7.3, 5.2, 1.0 Hz, 1H), 2.41 (d, J = 7.6 Hz, 1H), 2.33 (s, 1H), 2.19-2.23 (m, 7H), 1.61-1.71 (m, 2H), 1.46-1.51 (m, 4H), 1.23 (s, 26H), 0.86 (d, J = 6.6 Hz, 3H); ^{13}C NMR (101 MHz) δ 179.59, 173.16, 152.02, 145.52, 139.81, 119.31, 115.02, 77.62, 77.58, 65.42, 65.32, 37.32, 34.48, 32.00, 29.72, 29.57, 29.43, 29.29, 29.19, 29.00, 28.94, 28.85, 28.44, 25.36, 24.95, 22.77, 19.28, 14.21. HRMS (ESI-TOF) $[M+H]^+$ Calcd for $[C_{33}H_{50}NO_2]$ 492.3836, found 492.3829.

_N_-(4-Fluorophenyl)pentacosa-10,12-diynamide (DA 7)

^{1}H NMR (400 MHz, $CDCl_3$) δ 7.64 (s, 1H), 7.45 (dd, J = 9.1, 4.8 Hz, 2H), 6.95 (t, J = 8.7 Hz, 2H), 2.30 (t, J = 7.6 Hz, 2H), 2.21 (t, J = 6.9 Hz, 4H), 1.63-1.70 (m, 2H), 1.43-1.52 (m, 4H), 1.23-1.35 (m, 26H), 0.85 (t, J = 6.8 Hz, 3H); ^{13}C NMR (101 MHz) δ 171.76, 160.54, 158.13, 134.10, 121.87, 121.79, 115.70, 115.48, 77.77, 77.56, 65.43, 65.33, 37.60, 32.00, 29.72, 29.57, 29.43, 29.26, 29.18, 28.95, 28.82, 28.44, 28.34, 25.68, 22.77, 19.28, 19.25, 14.22. HRMS (ESI-TOF) $[M+H]^+$ Calcd for $[C_{31}H_{47}FNO]$ 468.3636, found 468.3667.

_N_-(Naphthalen-1-yl)pentacosa-10,12-diynamide (DA 8)

^{1}H NMR (400 MHz, $CDCl_3$) δ 7.80 (s, 4H), 7.65 (d, J = 8.0 Hz, 1H), 7.46 (s, 2H), 7.39 (t, J = 7.6 Hz, 1H), 2.41 (t, J = 7.1 Hz, 2H), 2.20-2.23 (m, 4H), 1.71 (d, J = 6.5 Hz, 2H), 1.49 (t, J = 7.0 Hz, 4H), 1.30 (d, J = 40.7 Hz, 26H), 0.85-0.89 (m, 3H); ^{13}C NMR (101 MHz) δ 172.34, 134.17, 132.47, 128.72, 127.54, 126.23, 125.99, 125.89, 125.73, 121.47, 121.02, 77.73, 77.57, 65.45, 65.37, 37.53, 32.02, 29.74, 29.59, 29.45, 29.29, 29.20, 28.96, 28.86, 28.46, 28.39, 25.91, 22.80, 19.29, 14.24. HRMS (ESI-TOF) $[M+H]^+$ Calcd for $[C_{35}H_{50}NO]$ 500.3887, found 500.3878.

DA 1

^{1}H NMR

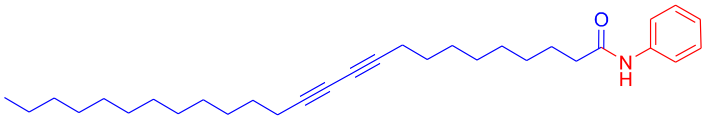

_N_-Phenylpentacosa-10,12-diynamide

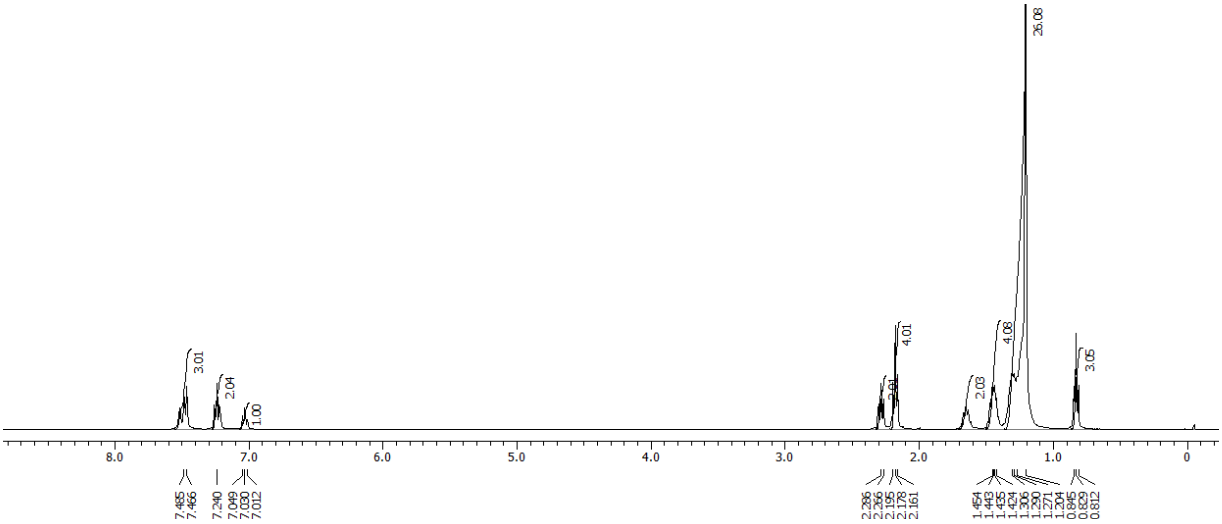

DA 1

^{13}C NMR

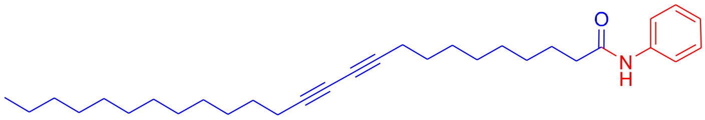

_N_-Phenylpentacosa-10,12-diynamide

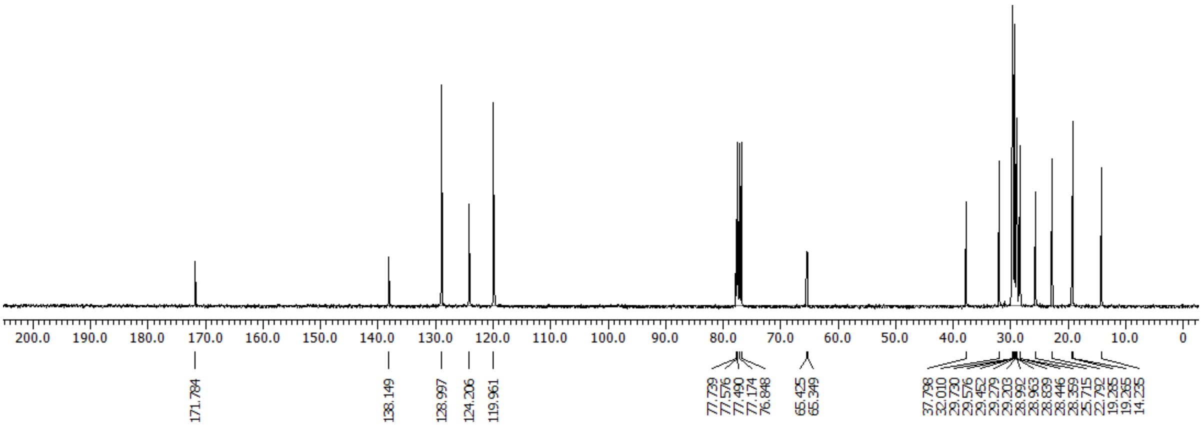

DA 1

HRMS

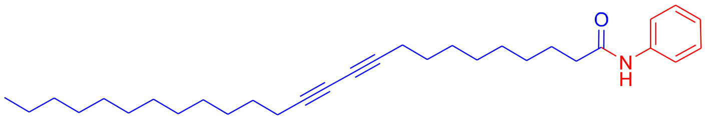

N-Phenylpentacosa-10,12-diynamide

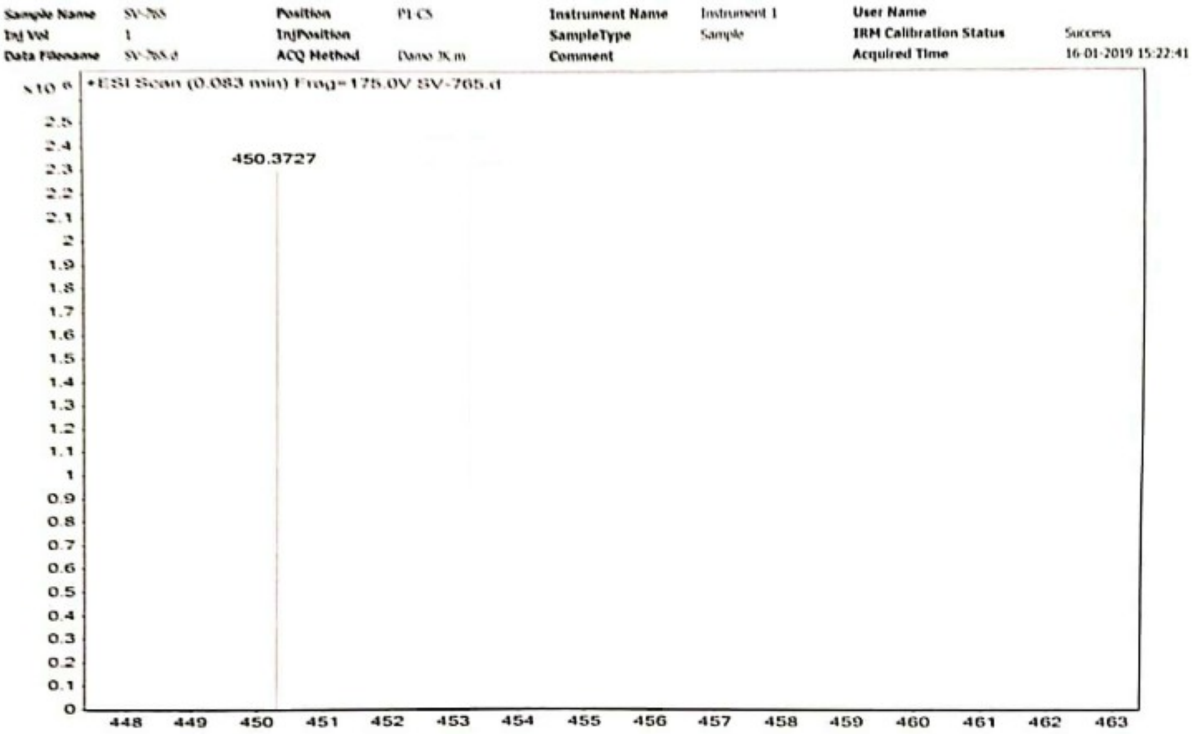

^{1}H NMR

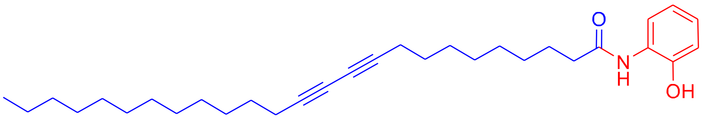

***N*-(2-hydroxyphenyl)pentacosa-10,12-diynamide**

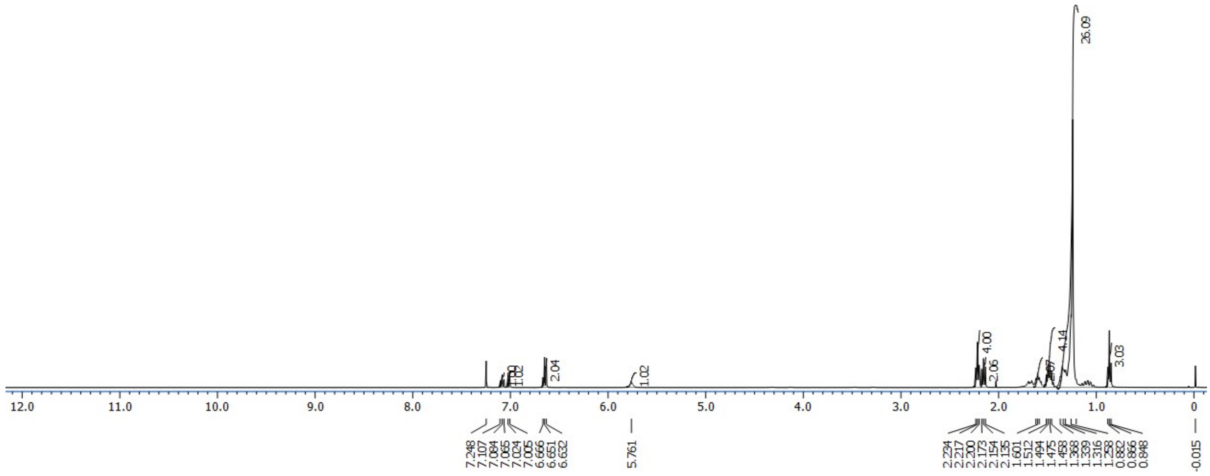

DA 2

^{13}C NMR

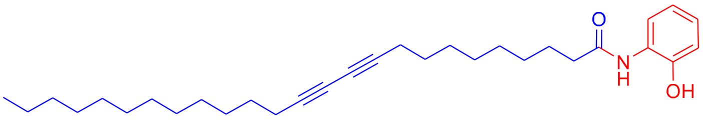

***N*-(2-hydroxyphenyl)pentacosa-10,12-diynamide**

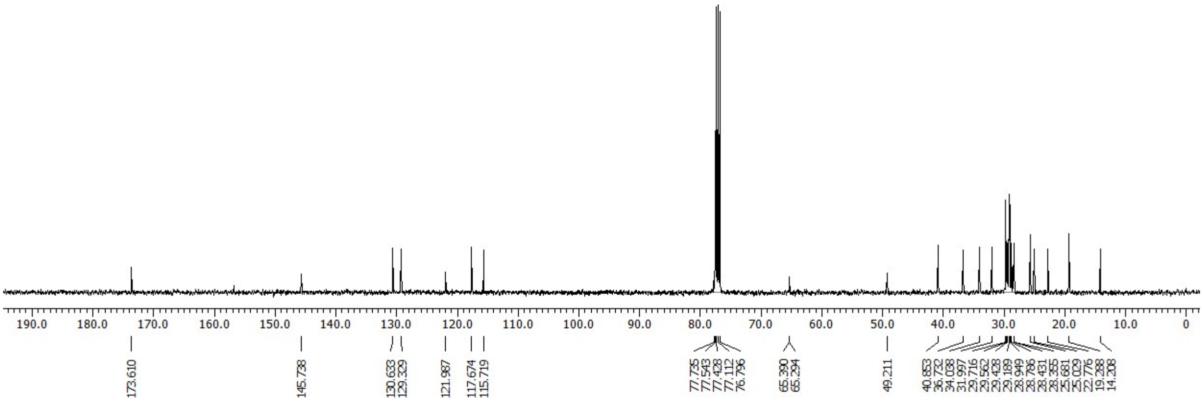

DA 2

HRMS

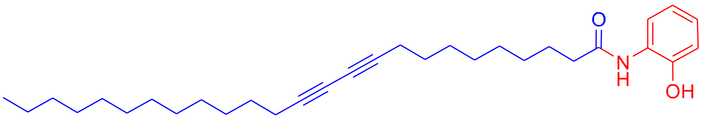

***N*-(2-hydroxyphenyl)pentacosa-10,12-diynamide**

Qualitative Compound Report

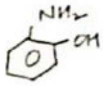

**Data File**	AM-AN-OH.d	**Sample Name**	AM-AN-OH
**Sample Type**	Sample	**Position**	P1-B9
**Instrument Name**	Instrument 1	**User Name**	
**Acq Method**	Damo JK.m	**Acquired Time**	29-04-2019 12:58:45
**IRM Calibration Status**	Success	**DA Method**	Default.m
**Comment**			

**Sample Group**		**Info.**
**Acquisition SW**	6200 series TOF/6500 series	
**Version**	Q-TOF B.05.01 (B5125.1)	

Compound Table

Compound Label	RT	Mass	Formula	MFG Formula	MFG Diff (ppm)	DB Formula
Cpd 2: C31 H47 N O2	0.086	465.3609	C31 H47 N O2	C31 H47 N O2	-0.55	C31 H47 N O2

Compound Label	*m/z*	RT	Algorithm	Mass
Cpd 2: C31 H47 N O2	466.3682	0.086	Find by Molecular Feature	465.3609

MFE MS Spectrum

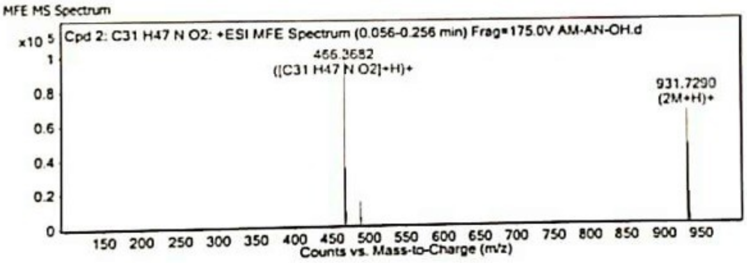

MFE MS Zoomed Spectrum

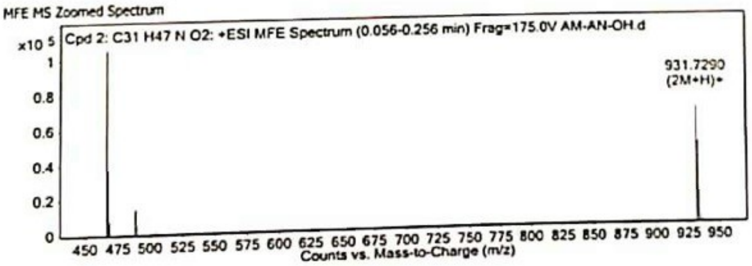

MS Spectrum Peak List

m/z	z	Abund	Formula	Ion
466.3682	1	105652.34	C31 H47 N O2	(M+H)+
467.3716	1	34580.3	C31 H47 N O2	(M+H)+
468.3749	1	8349.58	C31 H47 N O2	(M+H)+
488.3511	1	14103.26	C31 H47 N O2	(M+Na)+
489.3486	1	5077.69	C31 H47 N O2	(M+Na)+
504.3205	1	2283.39	C27 H43 N7	(M+K)+
931.729	1	67017.02		(2M+H)+
932.7317	1	46371.58		(2M+H)+
933.7351	1	17171.82		(2M+H)+
934.7373	1	4544.43		(2M+H)+

Scanned with CamScanner

— End Of Report —

^{1}H NMR

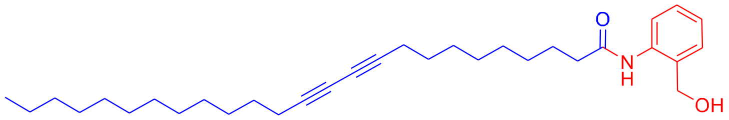

_N_-(2-(Hydroxymethyl)phenyl)pentacosa-10,12-diynamide

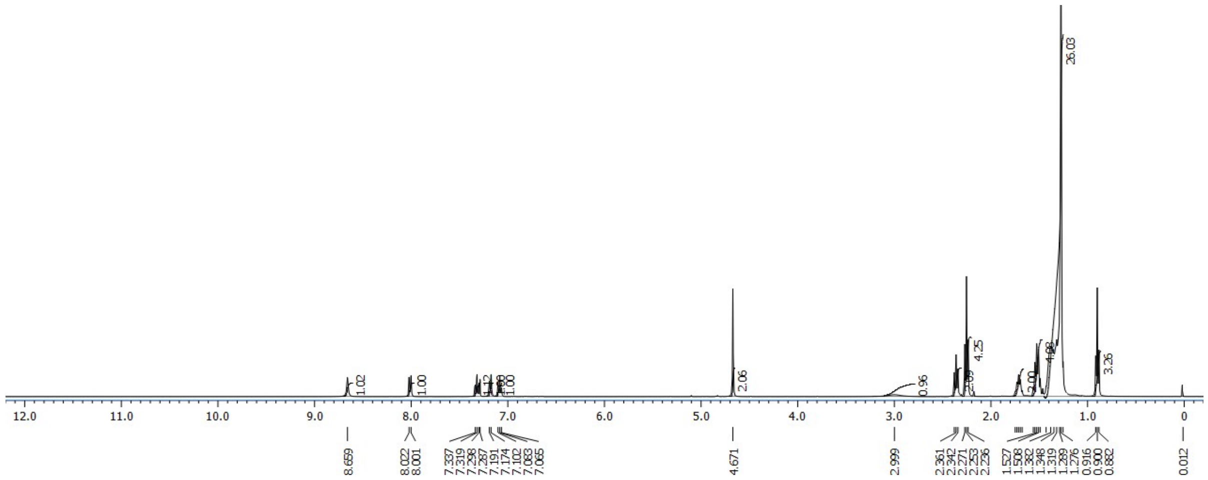

DA 3

^{13}C NMR

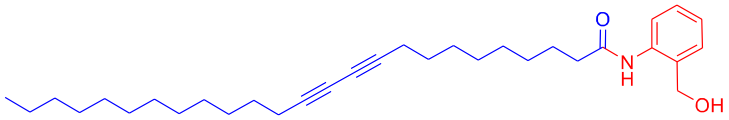

_N_-(2-(Hydroxymethyl)phenyl)pentacosa-10,12-diynamide

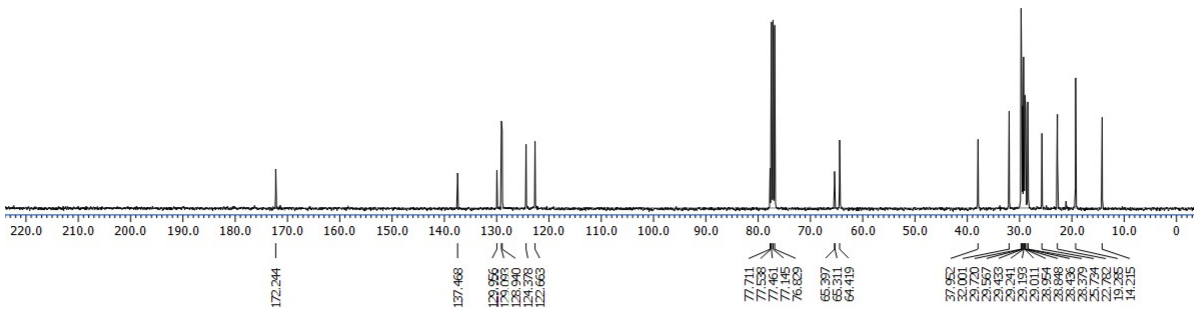

DA 3

HRMS

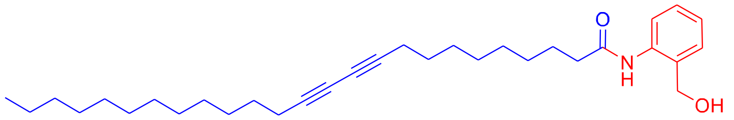

***N*-(2-(Hydroxymethyl)phenyl)pentacosa-10,12-diynamide**

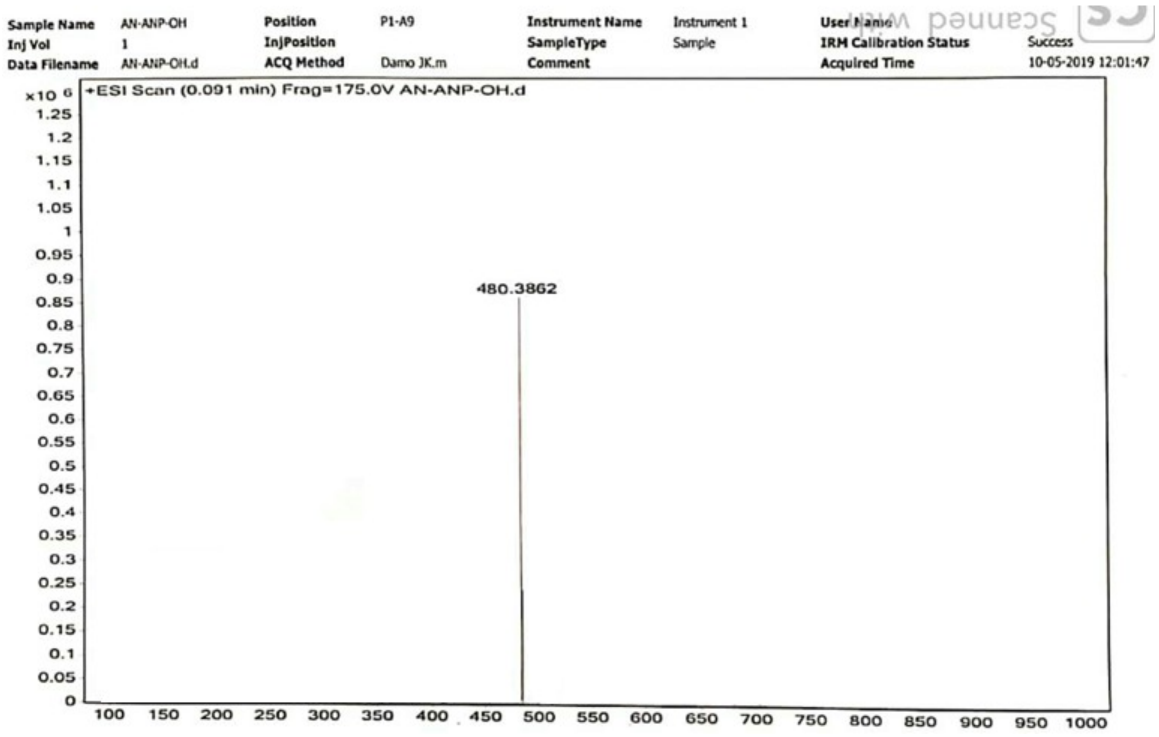

DA 4

^{1}H NMR

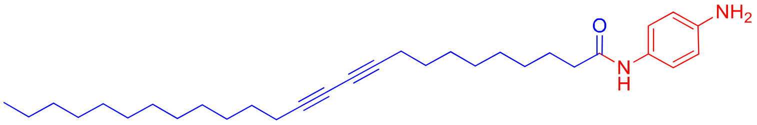

***N*-(4-aminophenyl)pentacosa-10,12-diynamide**

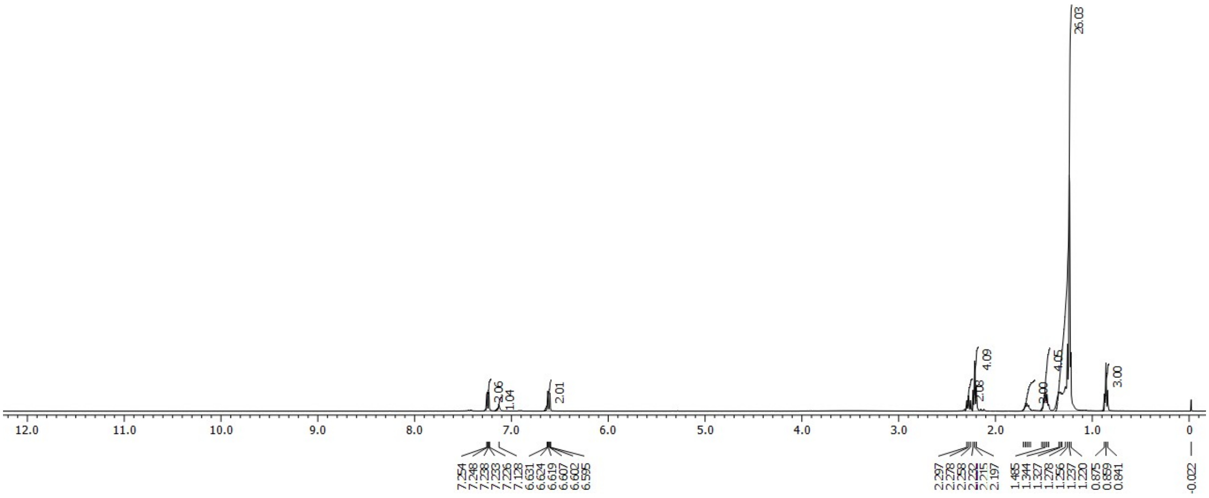

DA 4

^{13}C NMR

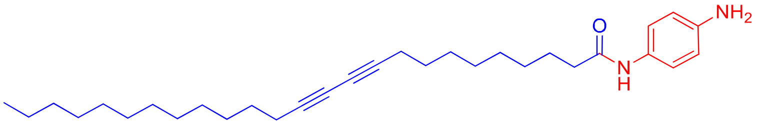

***N*-(4-aminophenyl)pentacosa-10,12-diynamide**

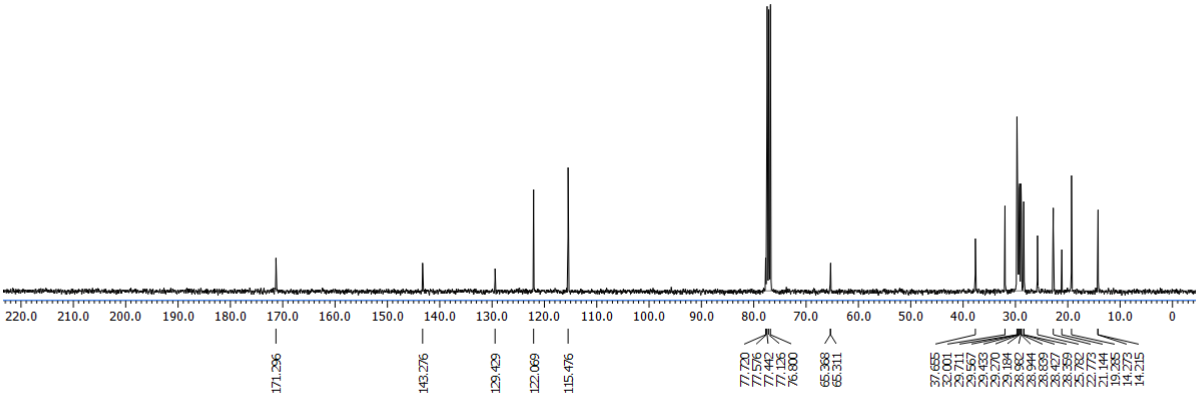

DA 4

HRMS

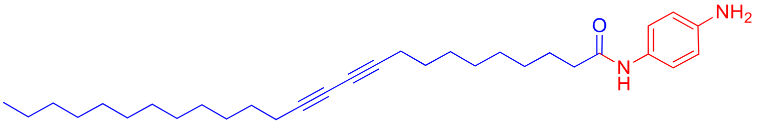

***N*-(4-aminophenyl)pentacosa-10,12-diynamide**

Qualitative Compound Report

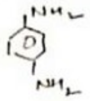

**Data File**	AM-1.4NH2.d	**Sample Name**	AM-1.4NH2
**Sample Type**	Sample	**Position**	P1-C9
**Instrument Name**	Instrument 1	**User Name**	
**Acq Method**	Damo JK.m	**Acquired Time**	08-05-2019 14:17:49
**IRM Calibration Status**	[redacted]	**DA Method**	Default.m
**Comment**			

**Sample Group**		**Info.**
**Acquisition SW**	6200 series TOF/6500 series	
**Version**	Q-TOF B.05.01 (B5125.1)	

Compound Table

Compound Label	RT	Mass	Formula	MFG Formula	MFG Diff (ppm)	DB Formula
Cpd 3: C31 H48 N2 O	0.095	464.3773	C31 H48 N2 O	C31 H48 N2 O	-1.37	C31 H48 N2 O

Compound Label	*m/z*	RT	Algorithm	Mass
Cpd 3: C31 H48 N2 O	465.3847	0.095	Find by Molecular Feature	464.3773

MFE MS Spectrum

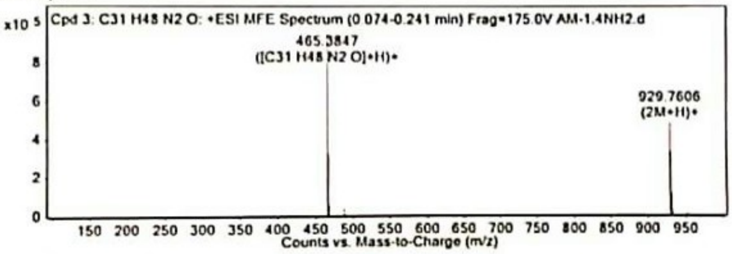

MFE MS Zoomed Spectrum

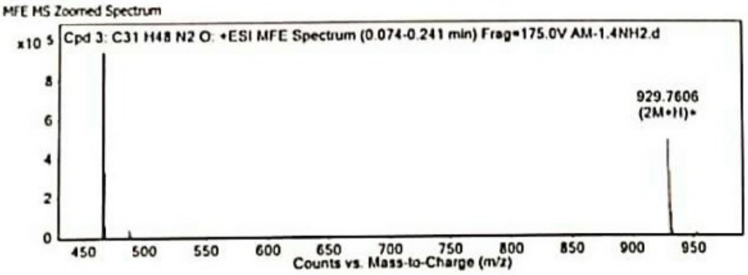

MS Spectrum Peak List

m/z	z	Abund	Formula	Ion
465.3847	1	954030.13	C31 H48 N2 O	(M+H)+
466.3877	1	320311.37	C31 H48 N2 O	(M+H)+
467.3906	1	55141.72	C31 H48 N2 O	(M+H)+
487.3655	1	37967.08	C31 H48 N2 O	(M+Na)+
488.3658	1	15014.84	C31 H48 N2 O	(M+Na)+
929.7606	1	486398.97		(2M+H)+
930.7638	1	331027.14		(2M+H)+
931.7666	1	111110.73		(2M+H)+
932.7691	1	26962.68		(2M+H)+
951.7411	1	13699.22		(2M+Na)+

DA 5

^{1}H NMR

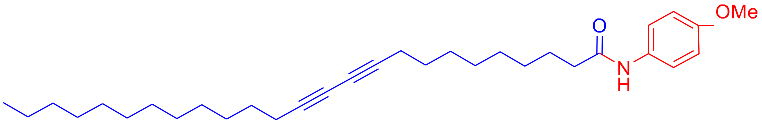

N-(4-Methoxyphenyl)pentacosa-10,12-diynamide

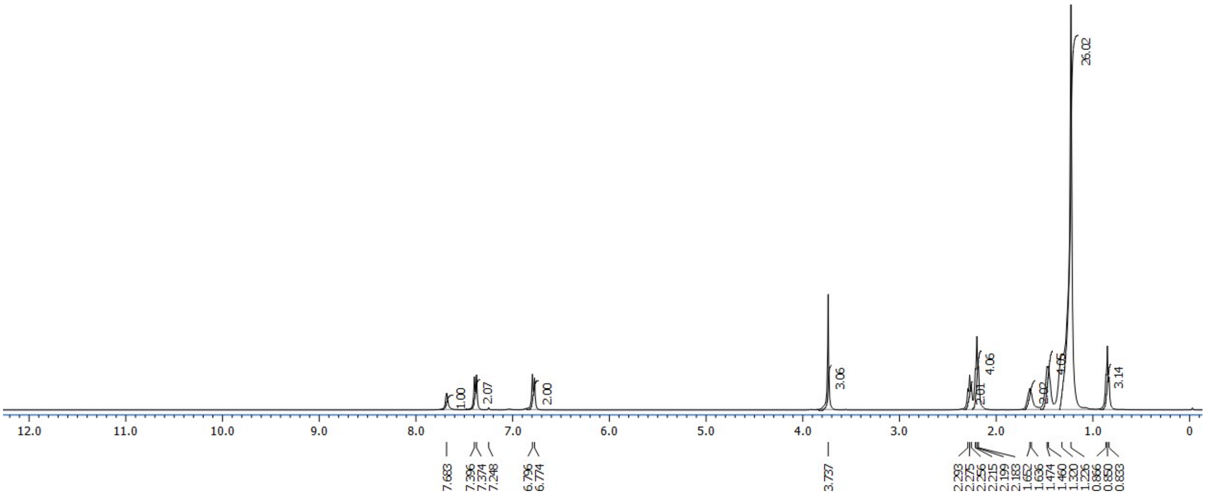

DA 5

^{13}C NMR

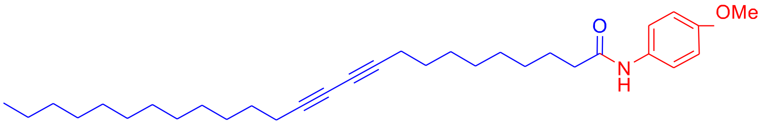

N-(4-Methoxyphenyl)pentacosa-10,12-diynamide

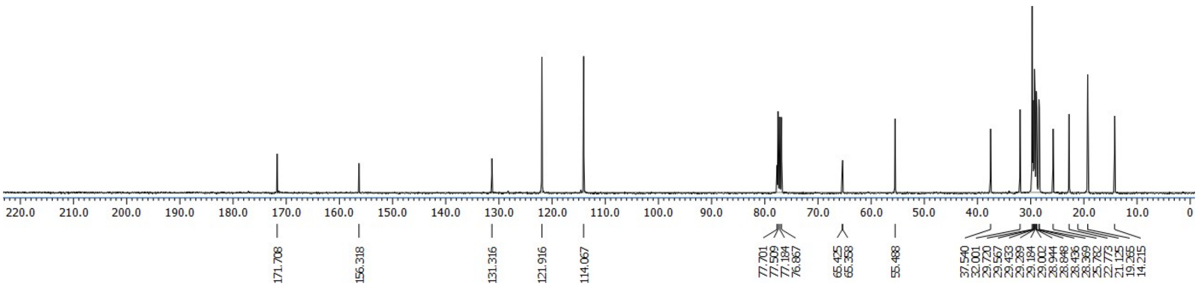

DA 5

HRMS

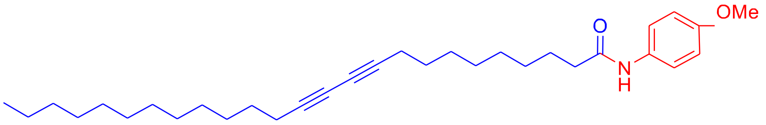

N-(4-Methoxyphenyl)pentacosa-10,12-diynamide

Qualitative Compound Report

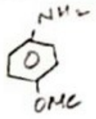

**Data File**	AM-AN-OME.d	**Sample Name**	AM-AN-OME
**Sample Type**	Sample	**Position**	P1-B8
**Instrument Name**	Instrument 1	**User Name**	
**Acq Method**	Damo JK.m	**Acquired Time**	29-04-2019 13:31:34
**IRM Calibration Status**	Success	**DA Method**	Default.m
**Comment**			

**Sample Group**		**Info.**
**Acquisition SW Version**	6200 series TOF/6500 series Q-TOF B.05.01 (B5125.1)	

Compound Table

Compound Label	RT	Mass	Formula	MFG Formula	MFG Diff (ppm)	DB Formula
Cpd 1: C32 H49 N O2	0.099	479.3776	C32 H49 N O2	C32 H49 N O2	-2.56	C32 H49 N O2

Compound Label	m/z	RT	Algorithm	Mass
Cpd 1: C32 H49 N O2	480.3848	0.099	Find by Molecular Feature	479.3776

MFE MS Spectrum

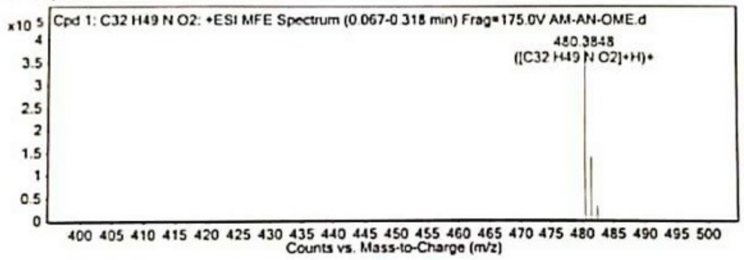

MFE MS Zoomed Spectrum

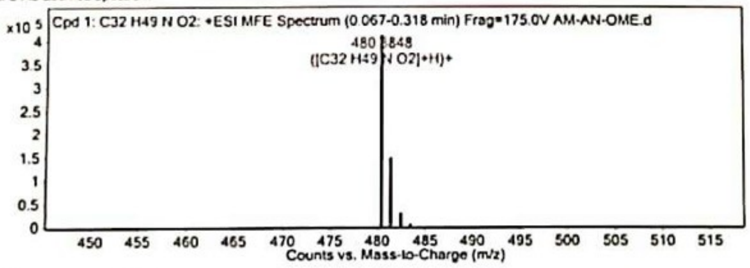

MS Spectrum Peak List

m/z	z	Abund	Formula	Ion
480.3848	1	413140.38	C32 H49 N O2	(M+H)+
481.3879	1	140254.24	C32 H49 N O2	(M+H)+
482.3928	1	30595.1	C32 H49 N O2	(M+H)+
483.3956	1	5261.55	C32 H49 N O2	(M+H)+

--- End Of Report ---

CS Scanned with CamScanner

DA 6

^{1}H NMR

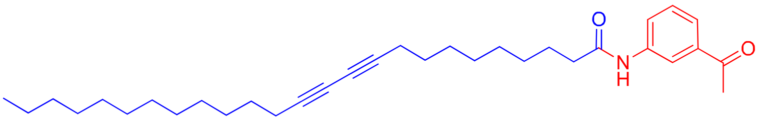

***N*-(3-Acetylphenyl)pentacosa-10,12-diynamide**

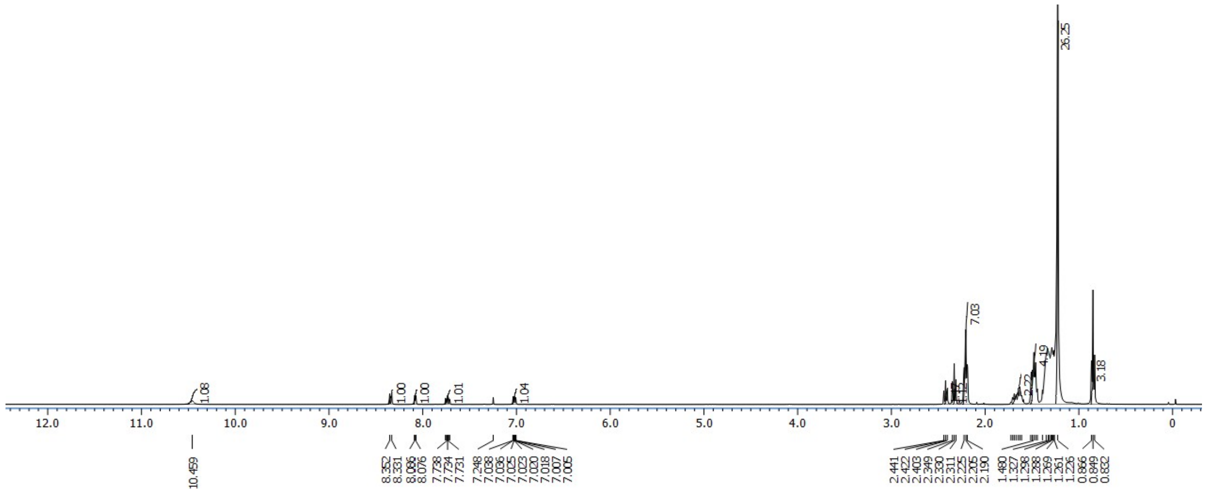

DA 6

^{13}C NMR

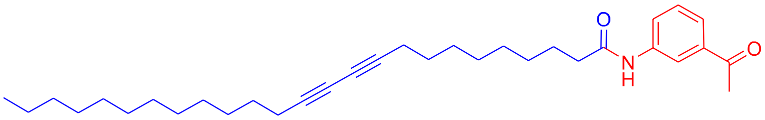

***N*-(3-Acetylphenyl)pentacosa-10,12-diynamide**

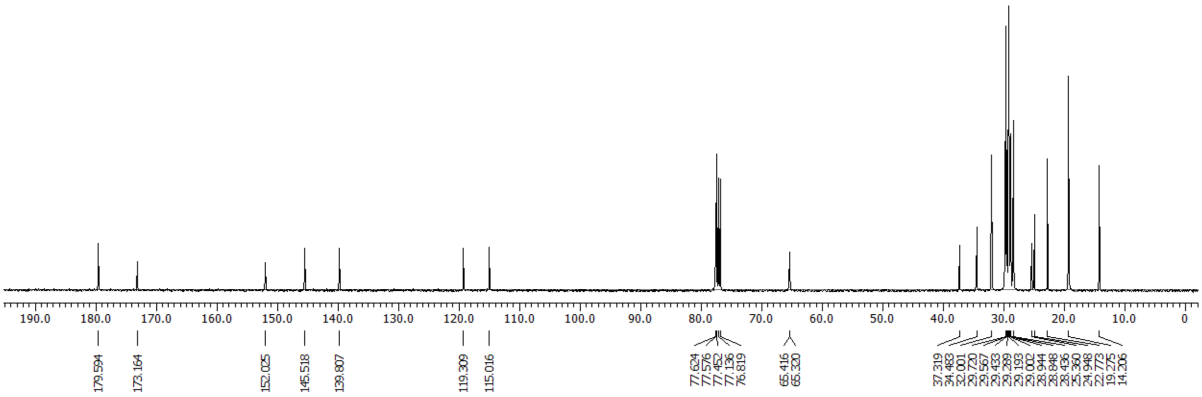

DA 6

HRMS

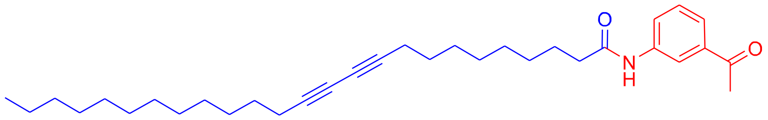

***N*-(3-Acetylphenyl)pentacosa-10,12-diynamide**

Qualitative Compound Report

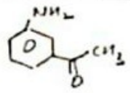

**Data File**	AM-AN-30CH.d	**Sample Name**	AM-AN-30CH
**Sample Type**	Sample	**Position**	P1-E3
**Instrument Name**	Instrument 1	**User Name**	
**Acq Method**	Damo JK.m	**Acquired Time**	09-05-2019 15:22:11
**IRM Calibration Status**	Success	**DA Method**	Default.m
**Comment**			

**Sample Group**		**Info.**
**Acquisition SW Version**	6200 series TOF/6500 series Q-TOF B.05.01 (B5125.1)	

Compound Table

Compound Label	RT	Mass	Formula	MFG Formula	MFG Diff (ppm)	DB Formula
Cpd 1: C33 H49 N O2	0.097	491.3746	C33 H49 N O2	C33 H49 N O2	3.49	C33 H49 N O2

Compound Label	*m/z*	RT	Algorithm	Mass
Cpd 1: C33 H49 N O2	983.7585	0.097	Find by Molecular Feature	491.3746

MFE MS Spectrum

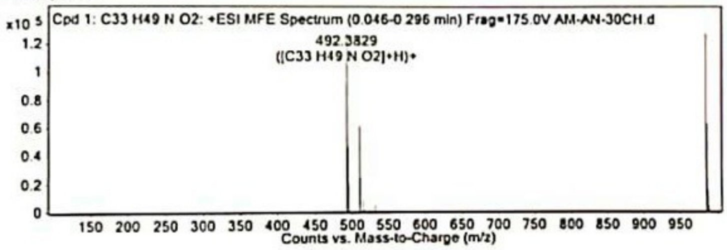

DA 7

^{1}H NMR

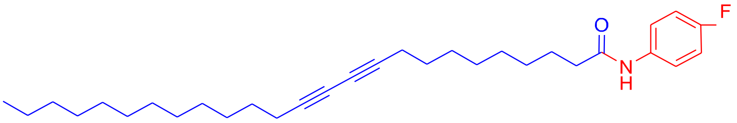

***N*-(4-Fluorophenyl)pentacosa-10,12-diynamide**

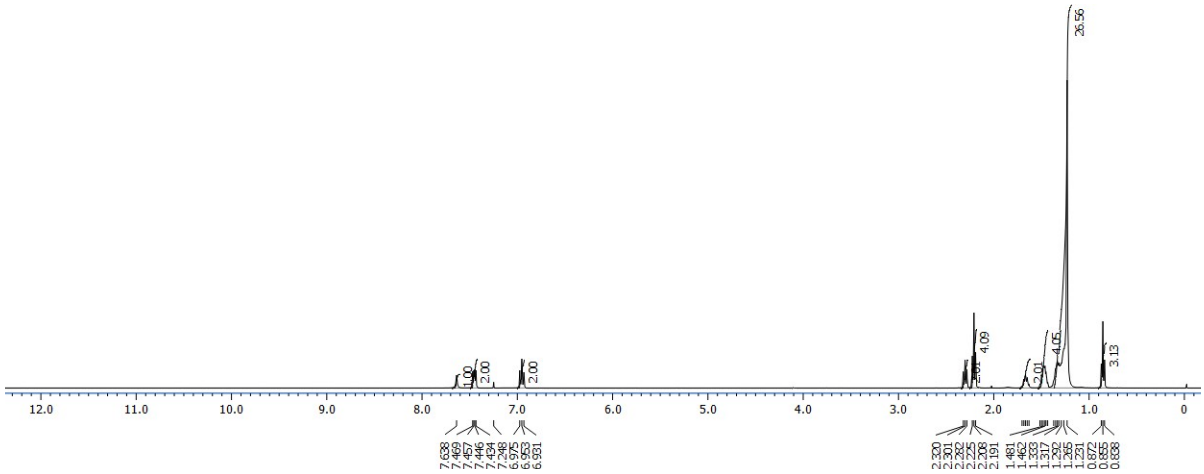

DA 7

^{13}C NMR

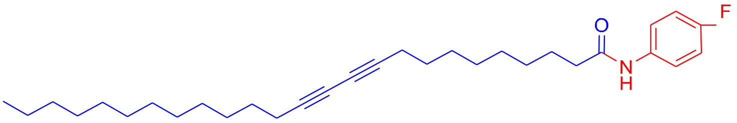

***N*-(4-Fluorophenyl)pentacosa-10,12-diynamide**

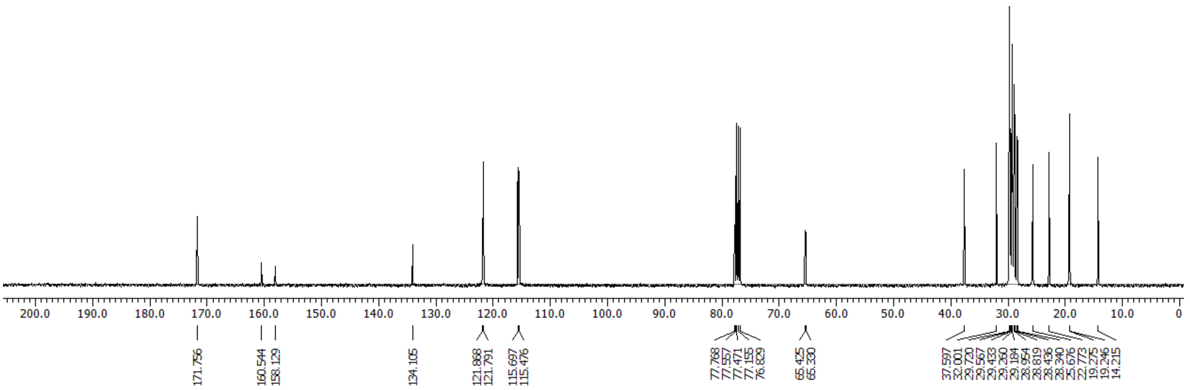

DA 7

HRMS

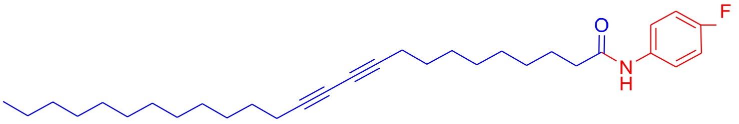

***N*-(4-Fluorophenyl)pentacosa-10,12-diynamide**

Qualitative Compound Report

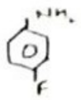

**Data File**	AM-AN-F.d	**Sample Name**	AM AN F
**Sample Type**	Sample	**Position**	P1 D9
**Instrument Name**	Instrument 1	**User Name**	
**Acq Method**	Damo JK.m	**Acquired Time**	29-04-2019 15:51:54
**IRM Calibration Status**	Success	**DA Method**	Default.m
**Comment**			

**Sample Group**		**Info.**
**Acquisition SW**	6200 series TOF/6500 series	
**Version**	Q-TOF B.05.01 (B5125.1)	

Compound Table

Compound Label	RT	Mass	Formula	MFG Formula	MFG Diff (ppm)	DB Formula
Cpd 1: C31 H46 F N O	0.084	467.3593	C31 H46 F N O	C31 H46 F N O	-6.31	C31 H46 F N O

Compound Label	*m/z*	RT	Algorithm	Mass
Cpd 1: C31 H46 F N O	468.3667	0.084	Find by Molecular Feature	467.3593

MFE MS Spectrum

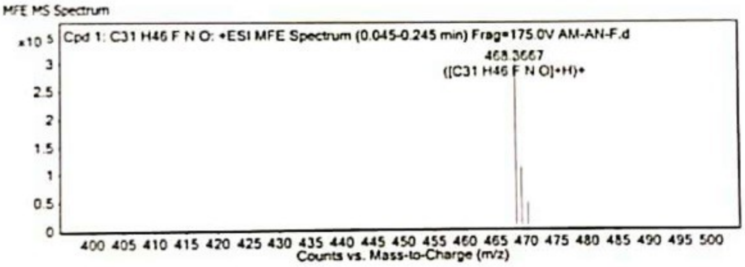

MFE MS Zoomed Spectrum

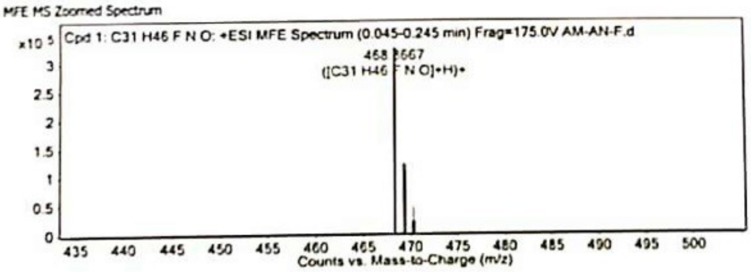

MS Spectrum Peak List

m/z	*z*	Abund	Formula	Ion
468.3667	1	325154.06	C31 H46 F N O	(M+H)+
469.3698	1	110667.44	C31 H46 F N O	(M+H)+
470.3723	1	47722.44	C31 H46 F N O	(M+H)+

--- End Of Report ---

DA 8

^{1}H NMR

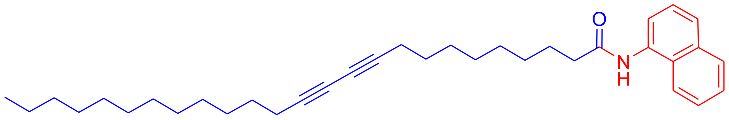

***N*-(Naphthalen-1-yl)pentacosa-10,12-diynamide**

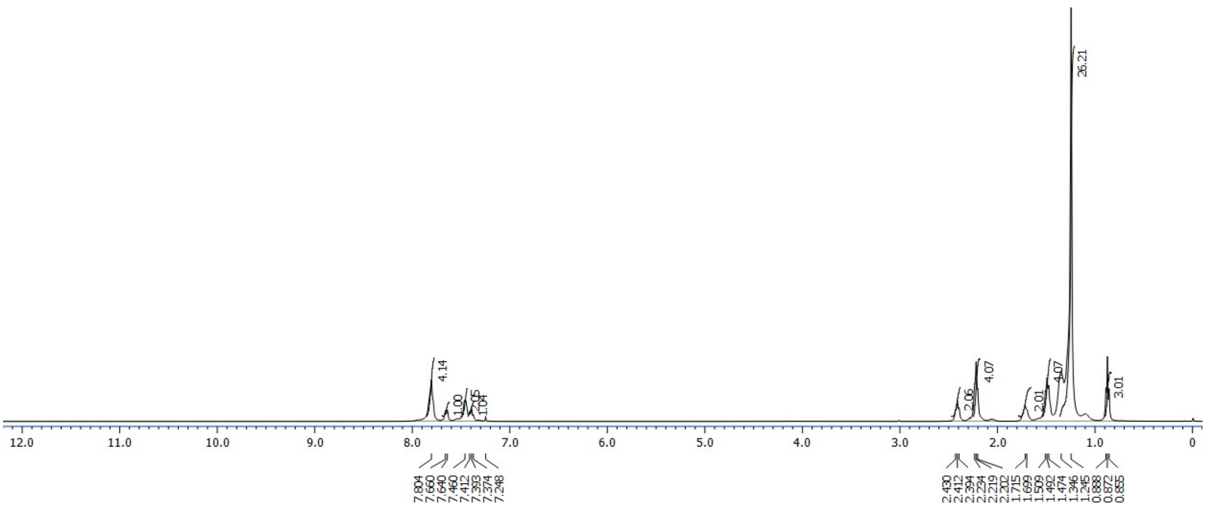

DA 8

^{13}C NMR

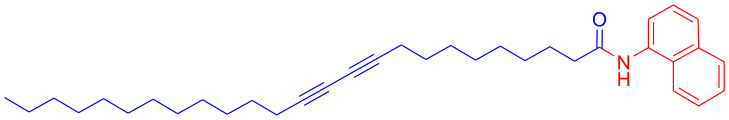

***N*-(Naphthalen-1-yl)pentacosa-10,12-diynamide**

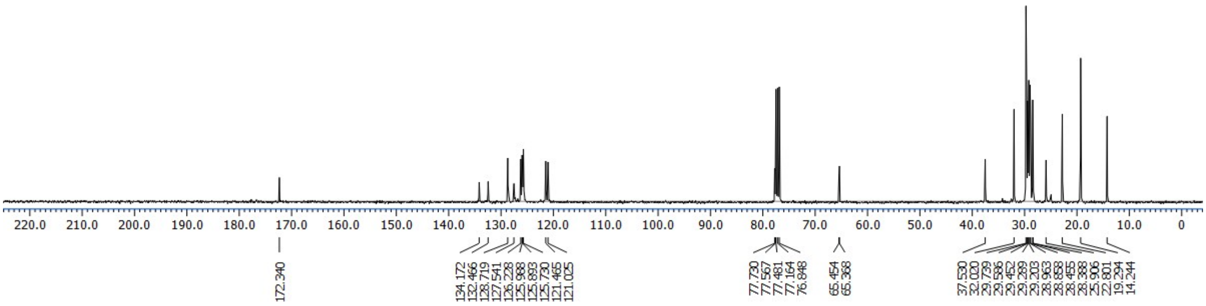

DA 8

HRMS

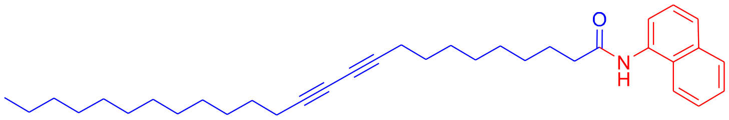

***N*-(Naphthalen-1-yl)pentacosa-10,12-diynamide**

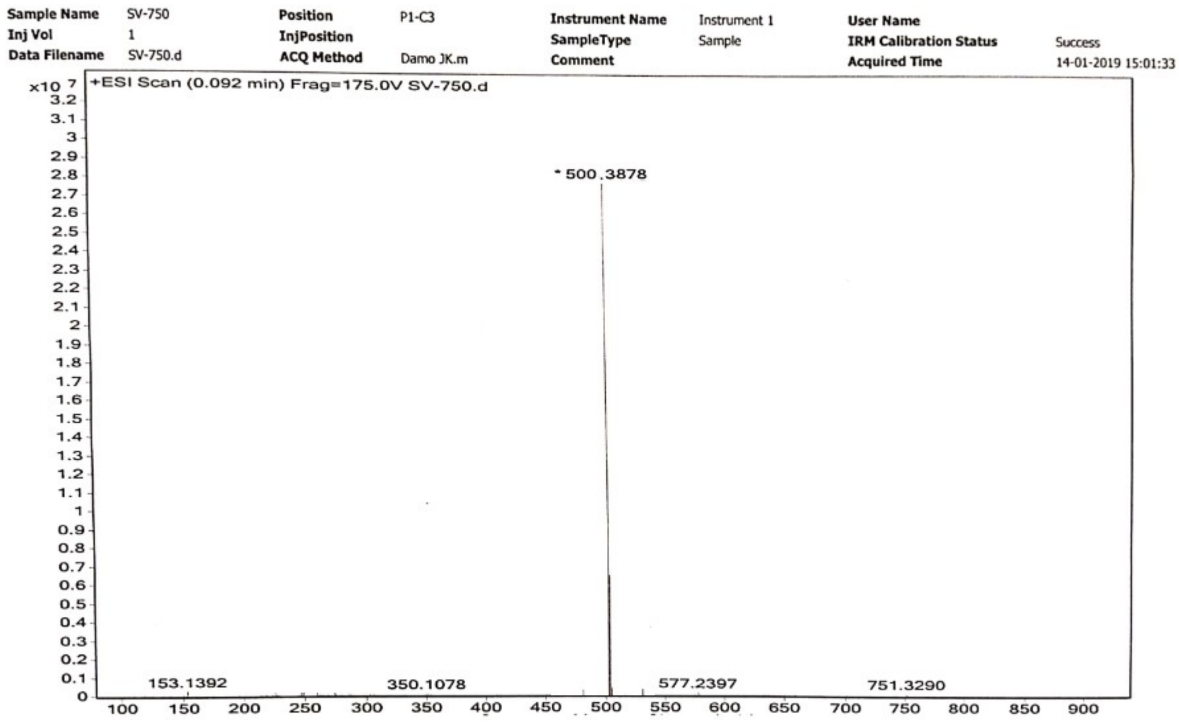

Supplement: Supplementary file 1 — Supplementary Information. [file 41598_2020_80115_MOESM1_ESM.pdf]
